# Targeted modulation of intestinal epithelial regeneration and immune response in ulcerative colitis using dual-targeting bilirubin nanoparticles

**DOI:** 10.7150/thno.87739

**Published:** 2024-01-01

**Authors:** Zewei Zhuo, Kehang Guo, Yujun Luo, Qi Yang, Huihuan Wu, Ruijie Zeng, Rui Jiang, Jingwei Li, Rui Wei, Qizhou Lian, Weihong Sha, Yuliang Feng, Hao Chen

**Affiliations:** 1 Department of Gastroenterology, Guangdong Provincial People's Hospital (Guangdong Academy of Medical Sciences), Southern Medical University, Guangzhou 510080, China.; 2 The Second School of Clinical Medicine, Southern Medical University, Guangzhou, China.; 3 School of Medicine, South China University of Technology, Guangzhou 510006, China.; 4 Department of Critical Care Medicine, The Fifth Affiliated Hospital of Zhengzhou University, Zhengzhou 450000, China.; 5 Department of Gastroenterology, The Third Affiliated Hospital of Sun Yat-Sen University, Guangzhou, 510630, China.; 6 Shantou University Medical College, Shantou 515041, China.; 7 Faculty of Synthetic Biology, Shenzhen Institute of Advanced Technology, Chinese Academy of Sciences, Shenzhen, China.; 8 Cord Blood Bank, Guangzhou Institute of Eugenics and Perinatology, Guangzhou Women and Children's Medical Center, Guangzhou Medical University, Guangzhou, China.; 9 State Key Laboratory of Pharmaceutical Biotechnology, The University of Hong Kong, SAR, China.; 10 Department of Pharmacology, School of Medicine, Southern University of Science and Technology, Shenzhen, Guangdong, 518055, China.; 11 Botnar Research Centre, Nuffield Department of Orthopaedics, Rheumatology and Musculoskeletal Sciences, University of Oxford Old Road, B4495, Headington, Oxford OX3 7LD, UK.

**Keywords:** ulcerative colitis, bilirubin, targeted therapy, intestinal stem cells, immune response

## Abstract

**Rationale:** The therapeutic benefits of bilirubin in the treatment of ulcerative colitis (UC) are considerable, whereas the underlying mechanism of bilirubin on UC remains unclear remains unexplored. In addition, the weak hydrophilicity and toxicity have limited its translational applications.

**Methods:** We have developed a colon dual-targeting nanoparticle, for orally delivering bilirubin through hydrogel encapsulation of hyaluronic acid (HA)-modified poly (lactic-co-glycolic acid) (PLGA) nanoparticles (HA-PLGA_Bilirubin_). Confocal microscopy and *in vivo* imaging were used to evaluate the uptake and the targeted property of HA-PLGA_Bilirubin_ in UC. Immunohistochemistry, immunofluorescence, and transcriptomic analyses were applied to examine the therapeutic effect and potential mechanism of HA-PLGA_Bilirubin_ in UC.

**Results:** Our results indicated that HA-PLGA_bilirubin_ can significantly enhance the release of bilirubin at simulated intestinal pH and demonstrate higher cellular uptake in inflammatory macrophages. Moreover, *in vivo* biodistribution studies revealed high uptake and retention of HA-PLGA_bilirubin_ in inflamed colon tissue of UC mouse model, resulting in effective recovery of intestinal morphology and barrier function. Importantly, HA-PLGA_bilirubin_ exerted potent therapeutic efficacy against ulcerative colitis through modulating the intestinal epithelial/stem cells regeneration, and the improvement of angiogenesis and inflammation. Furthermore, genome-wide RNA-seq analysis revealed transcriptional reprogramming of immune response genes in colon tissue upon HA-PLGA_bilirubin_ treatment in UC mouse model.

**Conclusion:** Overall, our work provides an efficient colon targeted drug delivery system to potentiate the treatment of ulcerative colitis via modulating intestinal epithelium regeneration and immune response in ulcerative colitis.

## Introduction

Ulcerative colitis (UC) is a chronic and recurrent gastrointestinal disease 1, dramatically impacts the quality of patients' life. Persistent and diffuse mucosal inflammation episodes, along with dysregulated mucosal immune response, are associated with the pathogenesis of UC 2, 3. Although the UC patients achieve symptom remission through the use of biologics, steroids, and other immunosuppressive agents 4, long-term administration of these medications may lead to serious complications such as hepatotoxicity, autoimmune diseases, and even malignancy 5-7. Therefore, there is an urgent need for an advanced therapeutic platform for the treatment of UC.

New candidate agents, such as potent antioxidant drug bilirubin, are being developed and have gained significant attention for their beneficial effects. As a hydrophobic byproduct of heme catabolism, bilirubin has been reported to possess powerful antioxidant effects 8-12 and anti-inflammatory properties 9, 13-15 in intestinal injury disease. Recent studies have also suggested that bilirubin may have immunomodulatory activity in colitis 15, 16, which further extends its clinical and translational value. However, the current understanding of the mechanisms underlying the effects of bilirubin in UC remains largely unexplored, and further investigation is necessary to fully elucidate its therapeutic potential.

Additionally, the hydrophobic nature and toxicity of bilirubin limit its delivery efficacy in vivo and hinder its clinical application 17. Previous literature has made progress in developing hyaluronic acid (HA)-based bilirubin nanoparticles for the treatment of colitis and acute kidney injury (AKI) 18, 19, demonstrating therapeutic potential in the laboratory. Nevertheless, their uncontrollable systemic diffusion and potential undesirable side effects limit their clinical application through oral administration 20, 21. Furthermore, gastric proteolytic enzymes and acidic conditions in the gastrointestinal tract (GIT) can cause partial degradation of HA, affecting its effectiveness in UC 22, 23. One way to overcome these challenges is by introducing a colon-targeted shell that protects HA from GIT degradation while enabling HA-bilirubin nanoparticles to specifically degrade in the colon. Hydrogels (chitosan/alginate) may be suitable candidates for the oral administration delivery of nanoparticles due to their biocompatibility and pH-responsive properties 24, although the introduction of hydrogels into HA-bilirubin nanoparticles has not been explored.

**Scheme 1:**
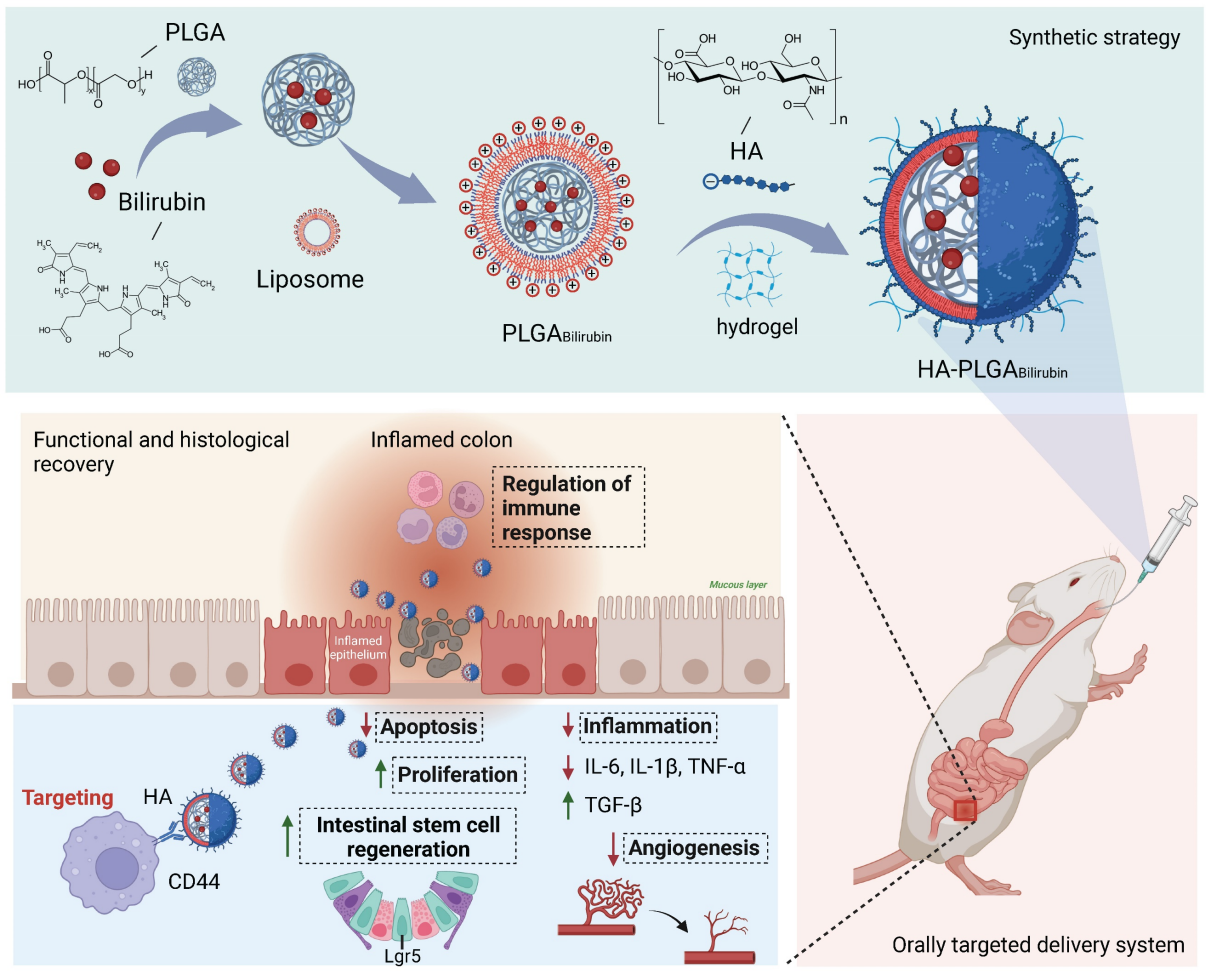
Schematic design of hydrogel encapsulating hyaluronic acid-modified PLGA nanoparticles (HA-PLGA_bilirubin_) for the targeted treatment of ulcerative colitis.

In this study, we aimed to develop a Hydrogel-HA-poly (lactic-co-glycolic acid) (PLGA) system for the oral delivery of bilirubin against UC. The system consists of hydrogel, PLGA, cationic liposome, and HA. The PLGA shell enhances the hydrophilicity of bilirubin, and the cationic liposome facilitates the adsorption of hyaluronic acid through electrostatic interactions, which enhances the targeting ability and therapeutic efficacy of the nanocomplex in inflammatory tissues. Moreover, with the assistance of the hydrogel, the HA-PLGA_bilirubin_ system can be effectively administered through oral gavage to achieve pH-responsive release and target inflamed colon tissues. Importantly, our study reveals novel recovery mechanisms of HA-PLGA_bilirubin_ in UC, including the modulation of colonic epithelial/stem cell regeneration, angiogenesis, and transcriptional reprogramming of immune response genes, thereby expanding our knowledge of the therapeutic mechanisms of bilirubin. The synthetic strategy, targeted delivery system, and histological recovery mechanism of the nanoparticles are summarized in Scheme 1. This study may enhance the bioavailability and therapeutic response of bilirubin, providing an effective oral targeted delivery option for the treatment of UC.

## Methods

### Preparation of HA-PLGA_bilirubin_

PLGA_bilirubin_ were fabricated using the emulsion-solvent evaporation method as described in previous reports. Briefly, 0.5 mL of PLGA (10 mg/mL), 0.1 mL of cationic liposome DOTAP (20 mg/mL), and 0.05 mL of bilirubin (10 mg/mL) were mixed in a chloroform solution as the organic phase. Then, 5 mL of deionized water was added for sonication in an ice bath for 2 min to generate oil-in-water (O/W) droplets. To remove excess chloroform, the solution was evaporated under rotation after the completion of the reaction. Subsequently, the supernatant containing PLGA_bilirubin_ was recovered by centrifugation for 30 min at 2000 rpm and 4 °C, and the excess free bilirubin was removed. To determine the encapsulation efficacy, the collected PLGA_bilirubin_ was lyophilized, dissolved in DMSO, and the concentration of bilirubin was then measured using a UV-vis-NIR spectrophotometer (Shimadzu UV-2600, Japan). The encapsulation efficiency of bilirubin was calculated as follows:



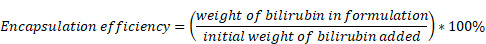



Afterward, HA solutions were added to the PLGA_bilirubin_ and mixed, followed by centrifugation. HA-PLGA_bilirubin_ were fabricated through self-assembly driven by electrostatic adsorption. To enhance stability, HA-PLGA_bilirubin_ can further cross-link with a hydrogel system to form an oral gel, as previously described 25. 12 μL of HA-PLGA_bilirubin_, 20 μL of calcium chloride (140 nM), and 8 μL of alginate (7.5 mg/mL) were mixed well and allowed to generate gelation. Blank HA-PLGA-lip nanoparticles were prepared using the same method without the addition of bilirubin.

### Characterization

The UV-vis absorption spectrum of free bilirubin and the prepared nanoparticles was measured using a UV-vis-NIR spectrophotometer. The size distribution and zeta potential of above nanoparticles were determined by Dynamic light scattering (DLS, Malvern Nano ZS90, British). The detailed structural analysis of the materials using Fourier Transform Infrared Spectroscopy (FT-IR). The morphology of the nanoparticles was analyzed by transmission electron microscopy (TEM, JEOL-2100, Japan). The Scanning Electron Microscopy (SEM) was used to investigate the particle size and morphology of HA-PLGA_Bilirubin_ hydrogel under different pH conditions, including simulated colonic fluid (SCF, PH=7.4), simulated intestinal fluid (SIF, pH 6.5), and simulated gastric fluid (SGF, PH=1.2). Scanning Electron Microscopy with Energy Dispersive X-ray Spectroscopy (SEM-EDS) was used to confirm that bilirubin was loaded inside the hydrogel.

### *In vitro* drug release

*In vitro* drug release experiments were conducted in modified enzymatic pH buffer media using simulated gastrointestinal fluids, as previously described 26. HA-PLGA_bilirubin_ hydrogel was added to 1 mL of simulated gastrointestinal fluid (SGF, pH=1.2), simulated intestinal fluid (SIF, pH=6.5), and simulated colon fluid (SCF, pH=7.4) at 0, 20, 40, 60, 120, 180, 240, and 300 min. After centrifugation at 2000 rpm, the supernatant containing nanoparticles was collected and subsequently lyophilized. Chloroform solution was added to dissolve the nanoparticles, and the concentration of free bilirubin was quantified by measuring its absorbance under ultraviolet light in a microplate. To observe the impact of hydrogel and non-hydrogel at different pH conditions on drug release, nanoparticles encapsulated in hydrogel and nanoparticles encapsulated in non-hydrogel were placed separately in SGF, SIF, and SCF at time points of 0 min, 60 min, and 180 min, respectively. The subsequent steps remain the same as before.

### Cellular uptake

The cellular uptake of HA-PLGA_bilirubin_ was analyzed using fluorescence-activated cell sorter (FACS) and confocal laser scanning microscopy (CLSM, Nikon E-A1, Japan). To mimic an inflammatory state, RAW 264.7 macrophages were treated with 100 μg of LPS and 10 μg of INF-r in serum-free conditions to induce a M1 phenotype. After 24 h of induction, DID-loaded nanoparticles were added to the cells for 4 h in serum-free conditions, and flow cytometry was performed to assess the uptake of the DID-loaded nanoparticles. The experimental method involved assessing the percentage of cells containing DID-loaded nanoparticles by establishing a cutoff fluorescence intensity derived from a histogram of control cells. Subsequently, the uptake of the DID-loaded nanoparticles by M1-induced RAW 264.7 macrophages was observed using CLSM. Alexa Fluor 488 phalloidin and DAPI were used to stain the membrane and nucleus of the cells, respectively.

### 3-(4,5-dimethylthiazol-2-yl)-2,5-diphenyltetrazolium bromide (MTT) assay

Cells were initially inoculated into a 96-well plate, left to adhere overnight. For each well, add 20 µl of MTT solution and incubate for 4 h. Terminate the culture, and carefully remove the culture supernatant. Add 150 µl of DMSO to each well, and shake for 10 min to ensure complete dissolution of the crystals. Measure the optical density of each well at 490 nm using an enzyme-linked immunosorbent assay reader and record the results.

### Construction of mimic UC animal model

Six-week-old C57/BL/6 mice were purchased from Guangdong Medical Laboratory Animal Center. Animal care and experiments were carried out in accordance with and approved by the Ethics Review Committee of Guangdong Provincial People's Hospital (KY-Z-2021-021). All mice were acclimatized for one week and then randomly assigned to different treatment groups. To induce the colitis model, mice were provided with drinking water containing 3% Dextran Sulfate Sodium Salt (DSS) for 5-6 days, followed by normal water for 4-5 days. Healthy mice were given only normal water as a control group. The DSS-induced mice were randomly divided into five groups (3-5 mice per group), and then administered with 30 mg/kg of bilirubin 18, HA-PLGA-lip, PLGA_bilirubin_ (equivalent mass of bilirubin as in 30 mg/kg bilirubin), and HA-PLGA_bilirubin_ (equivalent mass of HA-PLGA-lip and bilirubin) were dosed on predetermined days via oral gavage. Body weight was measured daily and the disease activity index (DAI) was determined to quantify colitis severity and the degree of improvement as previously stated. After cessation of various treatment, mice were sacrificed by decapitation and the colon length was measured from cecum to anus and photographed for preservation. The colon tissue specimens of the distal section were harvested for hematoxylin and eosin (HE) and immunofluorescence staining. The remaining colon tissues were snap frozen and stored at -80 °C for transcriptome sequencing analysis and the concentrations of cytokines and MPO level detection.

### *In vivo* biocompatibility evaluation

Six-week-old C57/BL/6 mice were orally treated with saline (control) or HA-PLGA_bilirubin_ (30 kg/mL) for 7 days. Blood was collected from the retroorbital region of mice for biochemical indicators detection, and their organs (heart, liver, spleen, lungs, and kidneys) were removed for histopathological analysis after cervical dislocation. The following hematology values were determined by an automated hematology analyzer (model XT-2000iV, Sysmex, Lincolnshire, IL): alanine aminotransferase (ALT), aspartate aminotransferase (AST), Alkaline Phosphatase (ALP), UREA, Total Protein (TP), red blood cells (RBC), white blood cells (WBC), platelet (PLT), mean corpuscular hemoglobin (MCH), mean corpuscular volume (MCV). Fixed organs were embedded in paraffin and 4% buffered formalin and 70% alcohol. Sections were stained with H&E and examined under a microscope.

### Rhodamine-(Rho) and Cy5.5 labeled HA-PLGA_Bilirubin_

Rhodamine-N-hydroxy succinimide (Rho-NHS) or Cy5.5-NHS was prepared at a 1:100 molar ratio to HA-PLGA_Bilirubin_. In brief, Rho-NHS or Cy5.5-NHS and EDC (1.2 equiv) were combined and stirred in 2 mL of DMSO for 20 min at room temperature in the absence of light. Following this, 1 mg/mL HA-PLGA_Bilirubin_ was introduced. After allowing this reaction mixture to proceed for 2 h at room temperature, any unreacted Rho-NHS or Cy5.5-NHS was eliminated via dialysis for up to 12 h using molecular-porous membrane tubing. This was followed by three precipitations with acetone and the end product underwent lyophilization.

### Biodistribution

To evaluate the biodistribution of nanoparticles in the colon, normal and DSS-induced mice were orally treated with Cy5.5-labeled HA-PLGA_Bilirubin_, Bilirubin, PLGA_Bilirubin_, and HA-PLGA_Bilirubin_. After oral delivery, mice were imaged using an AniView100 small animal living imaging system (BLT, China) at 4, 8, and 24 h. After 24 h, mice were sacrificed, and the heart, liver, spleen, lung, kidney, and colon were harvested for *ex vivo* imaging. Fluorescence intensity detection was analyzed using AniView100 Living Imaging software from BLT company. *In vivo* pharmacokinetic assessments were conducted using noncompartmental methods 27 to determine the maximum observed plasma concentrations (Cmax) and areas under the plasma concentration-time curve (AUC) from 0 to 72 h after various treatments.

### Flow Cytometry

The colon tissue was digested into a single-cell suspension. Subsequently, the following extracellular antibodies were added: Recombinant Alexa Fluor® 488 anti-EpCAM Antibody, Recombinant APC anti-Ly6G Antibody, PerCP/cyanine 5.5-conjugated anti-F4/80 Antibody, phycoerythrin (PE)-conjugated anti-CD11c Antibody, and Brilliant Violet 605™ anti-CD11b Antibody. The cells were incubated at room temperature in the dark for 30 min to immunostain cell surface markers. After surface molecule staining, the cells were resuspended in fixation/permeabilization solution and incubated in the dark for 15 min. Flow cytometry analysis was performed using a BD Celesta system, and the data were analyzed using FlowJo software.

### HE staining

A 4% paraformaldehyde solution was used to fix the colon tissues, followed by embedding them in paraffin. The paraffin-embedded specimens were then cut into 5 μm thick sections. Deparaffinization of the sections was performed using xylene, followed by hydration with gradient alcohol according to standard procedures 28. After washing the sections with distilled water, the nucleus of the sections was stained with hematoxylin for 10-15 min, followed by counterstaining with eosin for 2-5 min. Tissue morphology, including mucosal architecture changes, infiltration of inflammatory cells, goblet cell numbers, villus height, alterations in crypt drop out, and surface epithelial cell hyperplasia, was observed under light microscopy to evaluate the degree of intestinal mucosa damage and recovery in a double-blinded manner.

### Intestinal permeability assessment

Intestinal permeability assay was performed to assess the intestinal barrier functions of these mice through oral administration of 4 kDa FITC-dextran, as previously described 18. Briefly, mice with various treatment were fasted for 4 h, followed by oral administration of FITC-dextran (0.6 mg per gram of body weight). After 3 h, blood samples were collected via retro-orbital bleed, and the levels of FITC-dextran in the blood were measured using fluorescence, with excitation at 485 nm and emission at 520 nm.

### Immunohistochemical staining

After deparaffinization and hydration following the above procedures, tissue sections were subjected to antigen retrieval using 10 mM citrate acid under high pressure for 20 min. Subsequently, the sections were blocked with 10% goat serum for 60 min. After washing with a wash buffer, primary antibodies against PCNA (#13110, Cell Signaling Technology (CST)) were added to the sections and incubated overnight at 4 °C. Following this, the sections were incubated with biotin-labeled secondary antibodies for 30 min at 37 °C, followed by incubation with streptavidin-coupled horseradish peroxidase (HRP) for an additional 30 min at 37 °C. Color development was achieved using diaminobenzidine (DAB) chromogen for 3 min, and hematoxylin was finally used as a counterstain for 2-3 s. PCNA-positive nuclei in the crypt were visualized under a light microscope after the immunohistochemical procedures.

### Immunofluorescence staining

The tissue sections were deparaffinized, hydrated, subjected to antigen retrieval, and blocked with 10% donkey serum following the above procedures. For CD31 staining, primary antibodies against CD31 (1:1000, Company) were incubated overnight at 4 °C. The next day, the sections were incubated with a fluorescence secondary antibody, Alexa Fluor®594 donkey anti-rat IgG (H+L) (1:400, Life Technologies, A21209), for 30 min at 37 °C. After rinsing with tris buffered saline (TBS), the sections were stained with DAPI solution (Roche, 216276) for nuclear staining. The sections were then sealed with an anti-fluorescence quencher and observed under a darkfield microscope.

### TUNEL staining

TUNEL staining was performed using the In Situ TUNEL Detection Kits (Roche Diagnostics GmbH). According to the manufacturer's instructions, tissue sections were pretreated and incubated with Proteinase K working solution for 15-30 min at 37 °C. After washing, the TUNEL reaction mixture was added and incubated for 60 min at 37 °C in a humidified and dark environment. DAPI solution was applied to stain the nuclei, and then the sections were analyzed under a fluorescence microscope.

### Enzyme-Linked Immunosorbent Assay (ELISA) analysis

ELISA kits for MPO, VEGF-A, TGF-β, IL-4, IL-1β, IL-6, TNF-α, and IFN-γ were purchased from CUSABIO company. According to the manufacturer's instructions, colon tissues were broken into small pieces and homogenized in PBS solution using a tissue homogenizer. After centrifugation at 5000 x g for 5 min at 4 °C, the supernatants were collected and stored at -20 °C or -80 °C.100 μl of standard and sample were added to each well and incubated for 2 h at 37 °C. After removing the liquid, 100 μl of Biotin-antibody (1x) was added to each well and incubated for 1 h at 37 °C. After washing, 100 μl of HRP-avidin (1x) was added to each well and incubated for 1 h at 37 °C. 90 μl of TMB Substrate was added to each well and incubated for 15 min at 37 °C in the dark. Then, 50 μl of Stop Solution was added to each well and incubated for 5 min. OD values were measured using a microplate reader at a wavelength of 450 nm, and standard curves were established to measure the concentrations of MPO, VEGF-A, TGF-β, IL-1β, IL-6, and TNF-α in each sample.

### Quantitative reverse transcription polymerase chain reaction (qRT-PCR)

Colon tissue was isolated from the intestinal tract and stored at -80 °C to prevent RNA degradation. Total RNA from the cells was extracted using the E.Z.N.A. Total RNA Isolation Kit (Omega, GA, USA). Reverse transcription to generate cDNAs was performed using the PrimeScript™ RT-PCR kit (TaKaRa, Otsu, Japan). Following the manufacturer's instructions for the Biorad CFX Connect (Bio-Rad Laboratories, CA, USA), quantitative real-time polymerase chain reaction (qRT-PCR) was carried out using SYBR Premix Ex Taq (TaKaRa, Otsu, Japan). The specific steps for qRT-PCR were conducted as previously described 29 to quantitatively measure the relative mRNA levels of tight junction-related genes (ZO-1 and Ocludin-1). The specific primer sequences used were as follows: ZO-1, 5′-CCA GCA ACT TTC AGA CCA CC-3′ (forward) and 5′-TTG TGT ACG GCT TTG GTG TG-3′ (reverse); Ocludin-1, 5′-GCT TAC AGG CAG AAC TAG ACG-3′ (forward) and 5′-TCT GCA GAT CCC TTA ACT TGC-3′ (reverse); β-actin, 5′-ATG GAA TCC TGT GGC ATC CAT-3′ (forward) and 5′-TCC TGC CATC CTG TCA GCA ATG-3′ (reverse).

### Western Blot

As the previous study described 30, proteins are extracted, separated on an SDS-PAGE gel, and transferred to a membrane. After blocking with 5% non-fat milk, the membrane is probed with a primary antibody specific to the target protein, followed by incubation with a secondary antibody. Washing steps remove unbound antibodies. The target protein is visualized using chemiluminescence, and its levels are quantified.

### Transcriptome sequencing analysis

As stated above, colon tissues were harvested from the following groups: normal mice group (Control), DSS-induced mice treated with PBS group (DSS), DSS-induced mice treated with PLGA_bilirubin_ (DSS+PLGA_bilirubin_), and DSS-induced mice treated with HA-PLGA_bilirubin_ (DSS+HA-PLGA_bilirubin_). The samples were stored at -80 °C, and RNA extraction and transcriptome sequencing analysis were performed. Data processing was conducted using Novomagic (https://magic.novogene.com).

### Statistical analysis

A statistical analysis was performed using Prism software (GraphPad Prism). Students t-test or Welch's t-test were used to analyze continuous variables. Statistical significance was considered at a *p*-value less than 0.05.

## Results

### Preparation and characterization of HA-PLGA_bilirubin_

**Figure 1:**
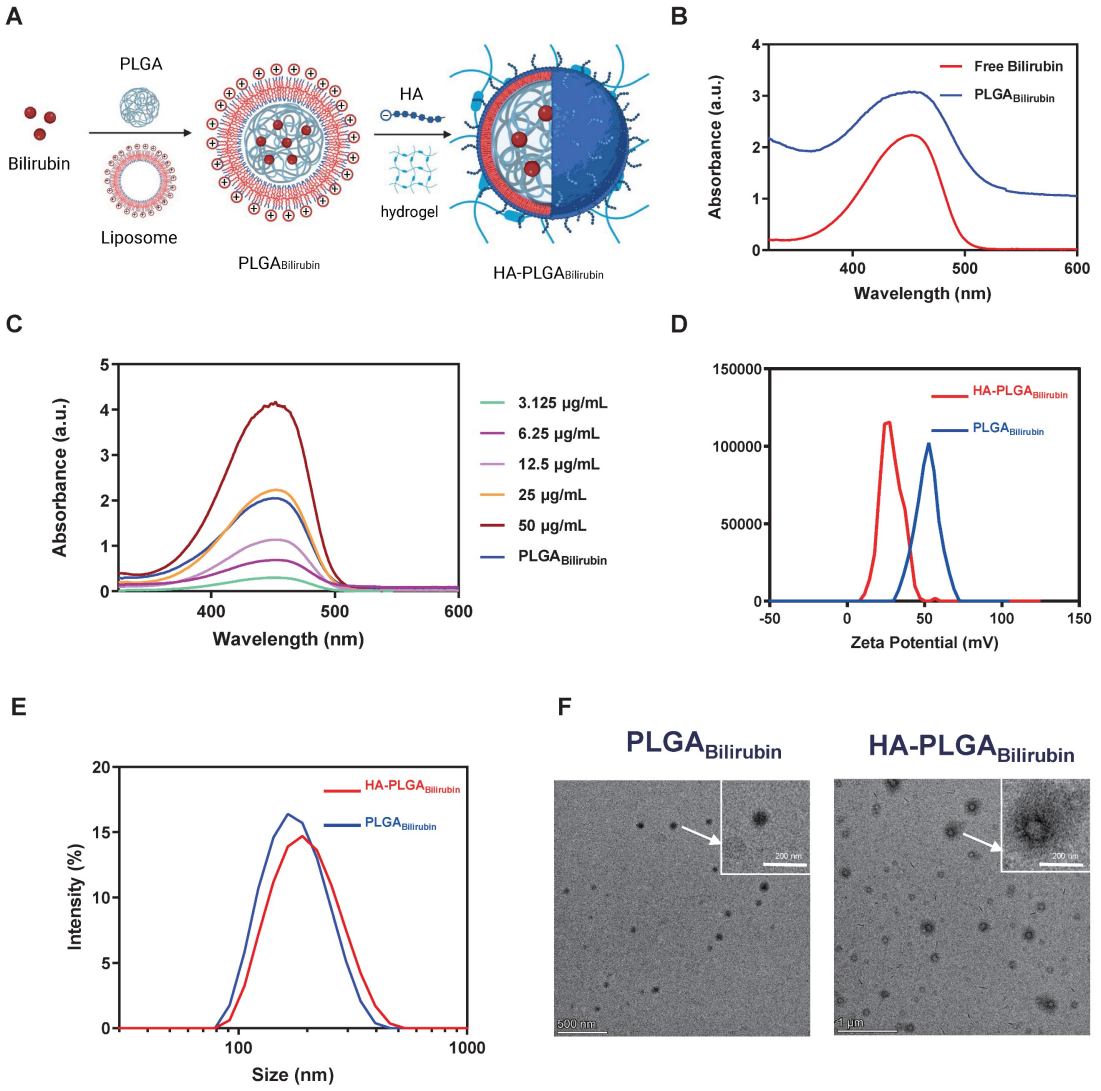
** Preparation and characterization of HA-PLGA_bilirubin_**. (A) Schematic design of the preparation of HA-PLGA_bilirubin_. (B) Absorption spectrum of PLGA_Bilirubin_. Absorptive peak becomes wide when the bilirubin was encapsulated into PLGA nanoparticles. (C) Drug-loading capacity of PLGA_Bilirubin_. The concentration of bilirubin in PLGA_Bilirubin_ was 23.8±1.3 μg/mL, and the drug encapsulation efficiency of PLGA_Bilirubin_ was 47.6±2.7%. (D) Zeta potential of HA-PLGA_Bilirubin_ and PLGA_Bilirubin_. (E) Size distribution of HA-PLGA_Bilirubin_ and PLGA_Bilirubin_. (F) Transmission electron microscopic (TEM) images of HA-PLGA_Bilirubin_ and PLGA_Bilirubin_.

We synthesized hydrogel-encapsulating HA-modified PLGA nanoparticles for the delivery of bilirubin (HA-PLGA_bilirubin_) using the double emulsion-solvent evaporation method and electrostatic adsorption interaction. The synthesis process is described in Figure 1A. The chloroform containing the mixture was removed by rotary evaporation to allow bilirubin to transfer to the PLGA hydrophobic core through the 'like dissolves like' principle. The half-width of the absorption peak broadened when the bilirubin was successfully coated with 1,2-dioleoyl-3-trimethylammonium-propane (DOTAP) and PLGA nanoparticles (Figure 1B). This change is probably because these substances interacted with each other, which caused the size range to increase 31 and affected the Localized Surface Plasmon Resonance (LSPR) 32, which, in turn, resulted in a broader peak in the absorption spectrum. Quantification of bilirubin loading in the DOTAP-PLGA nanoparticles was performed using UV to measure the drug loading yield and efficiency (Figure 1C). The bilirubin concentration was calculated to be 23.8±1.3 μg/mL based on the standard curve (Figure S1). According to the formulation of encapsulation efficiency, the encapsulation efficiency of bilirubin into DOTAP-PLGA nanoparticles was 47.6±2.7%, determining the effectiveness of the PLGA nanoparticles in entrapping the bilirubin. The nanoparticles were then characterized for size and zeta potential at each coating stage. The average particle size of PLGA_Bilirubin_ eventually stabilized at 163.91±1.96 nm, and the zeta potential stabilized at 53.26±2.73 mV by increasing the feeding of DOTAP (Figure S2; Figure S3). As HA is a negatively charged polysaccharide, it can be modified on the surface of PLGA_bilirubin_ by electrostatic adsorption, which is attributed to the cationic properties of cationic liposomes. When the PLGA_Bilirubin_ was modified with HA, the zeta potential decreased from 49.71±1.63 mV to 24.86±2.55 mV (Figure 1D), and the average particle size increased from 156.83±17.23 nm to 193.23±12.85 nm (Figure 1E). The polydispersity indices (PDIs) of the PLGA_Bilirubin_ and HA-PLGA_Bilirubin_ were <0.2, highlighting their narrow size distribution (Table S1). In fact, while DOTAP holds promise for delivering hydrophobic drugs 33-36, its highly positive charge can still pose issues related to hemolysis 37, 38. Here, we use the electrostatic adsorption of HA onto DOTAP liposomes to prepare HA-targeted nanoparticles. This approach helps neutralize the toxicity associated with cationic liposomes 39 and, by adsorbing HA, enables bilirubin nanoparticles to target CD44 ligands highly expressed in inflamed tissues 40. Transmission Electron Microscopy (TEM) images demonstrated that PLGA_Bilirubin_ and HA-PLGA_Bilirubin_ were vesicle-like nanoparticles with mean diameters of ~80 nm and ~120 nm, respectively (Figure 1F). Next, we conducted detailed structural analysis of the materials using Fourier Transform Infrared Spectroscopy (FT-IR) (Figure S4). The peaks at approximately 3407 cm^-1^ signify the existence of O-H functional groups. A distinctive absorption band at around 3420 cm^-1^ is observed in HA-PLGA_Bilirubin_, which correspond to their presence of multiple hydroxyl groups. The peaks observed in the range of 1693-1746 cm^-1^ are associated with the stretching vibrations of C=O bonds in the three different nanoparticles. Additionally, the peak at 1047 cm^-1^ can be attributed to the C-N stretching vibrations on PLGA_Bilirubin_ and HA-PLGA_Bilirubin_. Furthermore, the 1091 cm-1 peak corresponds to the stretching vibrations of ether bonds, which are present in PLGA_Bilirubin_ and HA-PLGA_Bilirubin_ but absent in Bilirubin. These results clearly indicate the structural distinctions between the three nanomaterials. In addition, after incubating the HA-PLGA_Bilirubin_ nanoparticles in both Phosphate-Buffered Saline (PBS) and Fetal Bovine Serum (FBS) for 8 days, their size remained essentially unchanged, demonstrating excellent stability in FBS (Figure S5).

### Uptake and drug release of HA-PLGA_bilirubin_

**Figure 2:**
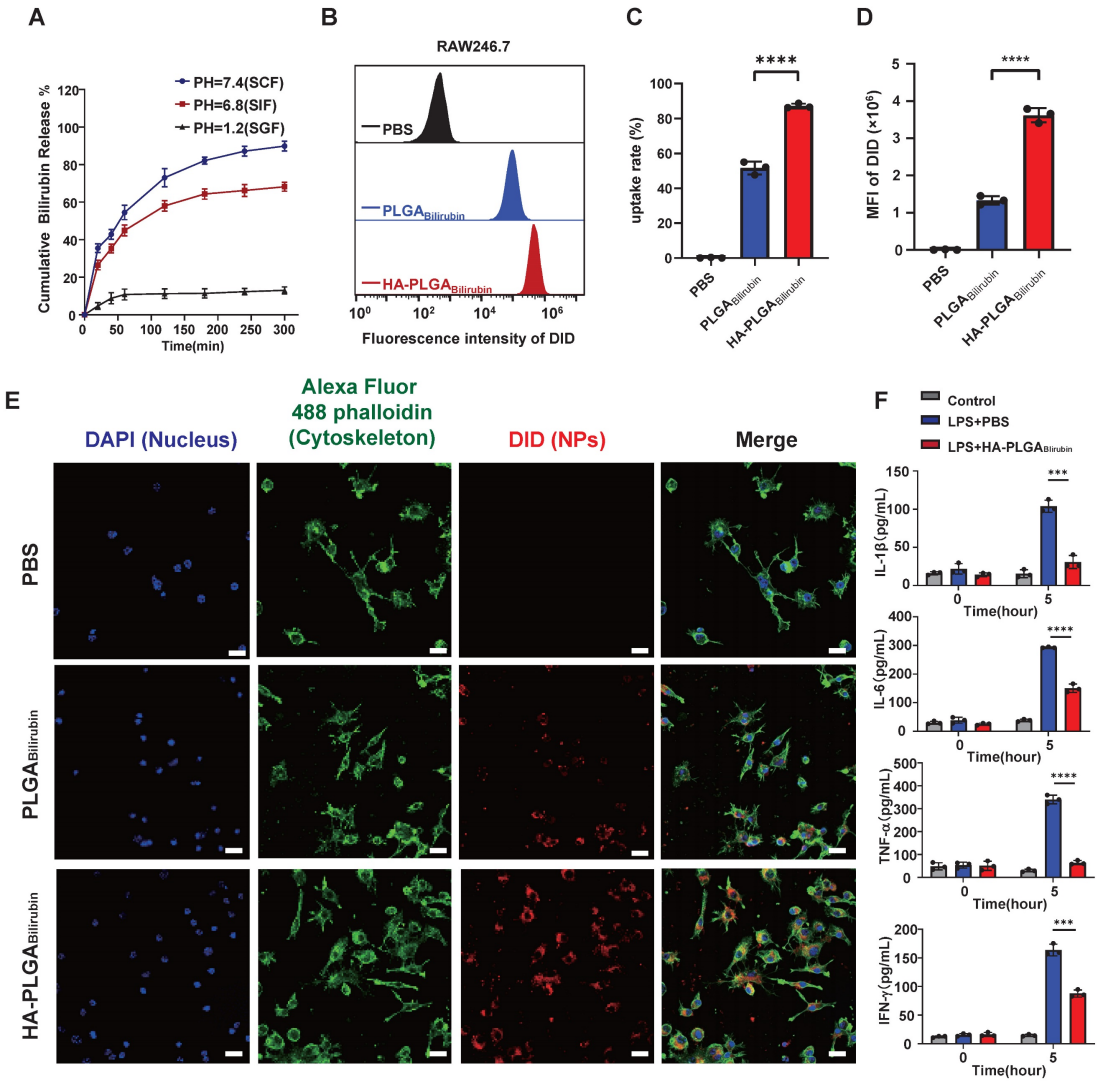
** Drug release, cellular uptake and pro-inflammatory cytokines downregulation of HA-PLGA_bilirubin_.** (A) Cumulative drug release profile of bilirubin in simulated gastrointestinal pH buffer media. (B) Flow cytometry histograms of uptake of PLGA_bilirubin_ and HA-PLGA_bilirubin_ by Raw 264.7 macrophages. (C) Cellular uptake percentages of PBS, PLGA_bilirubin_ and HA-PLGA_bilirubin_ by RAW 264.7 macrophages. (D) Mean fluorescent intensity (MFI) of Raw 264.7 macrophages treated with PLGA_bilirubin_ and HA-PLGA_bilirubin_. ****represents *p*<0.0001. (E) Confocal laser scanning microscopy (CLSM) image of RAW 264.7 macrophages treated with PLGA_bilirubin_ and HA-PLGA_bilirubin_. Scale bar, 20μm. (F) Relative protein levels of pro-inflammatory cytokines (IL-1β, IL-6, TNF-α, and IFN-γ) in LPS-induced RAW 264.7 macrophages.

The controllable release of bilirubin from HA-PLGA_bilirubin_ to the colonic tissues under specific pH conditions is critical for improving the bioavailability of the drug. Herein, we encapsulated the nanoparticles into a hydrogel composed of chitosan and alginate and observed the release of bilirubin in simulated gastrointestinal conditions. As shown in Figure 2A, the release of bilirubin at pH 7.4 (simulated colonic fluid, SCF) was higher than that at pH 1.2 (simulated gastric fluid, SGF), and slightly higher than PH 6.8 (simulated intestinal fluid, SIF). In addition, our results demonstrate that in simulated gastric fluid (SGF), nanoparticles without hydrogel encapsulation (HA-PLGA_Bilirubin_) achieved a release of 54.56%, which was 45.17% higher than that of HA-PLGA_Bilirubin_ hydrogel. HA-PLGA_Bilirubin_ within the hydrogel started substantial bilirubin release in SIF and gradually stabilized in SCF. These results clearly confirm the pH-responsive properties of the hydrogels (chitosan/alginate) for drug release (Figure S6). Furthermore, we conducted Scanning Electron Microscopy with Energy Dispersive X-ray Spectroscopy (SEM-EDS) analysis on both bilirubin and the HA-PLGA_Bilirubin_ hydrogel. As shown in Figure S7, the SEM-EDS results clearly demonstrate the composition of the materials. We found that carbon (C), nitrogen (N), and oxygen (O) are the primary elements in bilirubin. However, following the encapsulation of bilirubin within the hydrogel, we observed the appearance of calcium (Ca) and chlorine (Cl) components, accompanied by a decrease in the relative proportions of C, N, and O. This observation serves as supportive evidence that bilirubin was indeed loaded inside the hydrogel. The pH responsiveness of the hydrogel is attributed to the protonation and deprotonation of functional groups on the sodium alginate backbone and crosslinking agents, which result in changes in the polymer conformation and swelling behavior. Specifically, sodium alginate is a pH-sensitive, anionic polysaccharide that undergoes structural changes in response to pH variations. In acidic environments (e.g., stomach), the carboxyl groups in sodium alginate become protonated (-COOH), leading to a reduction in repulsive forces between the polymer chains.

This protonation allows the polymer chains to interact more strongly, resulting in the formation of a compact structure or "gel" that can withstand the harsh acidic conditions. Conversely, in more neutral to alkaline environments (e.g., the colon), the carboxyl groups lose their protons, transitioning to their anionic form (-COO-). This deprotonation increases electrostatic repulsion between polymer chains, causing the hydrogel to swell and disintegrate, thereby releasing the entrapped bilirubin. This pH-responsive behavior of sodium alginate facilitates the targeted delivery of bilirubin to the colonic area, where the conditions trigger gel disintegration and drug release. Additionally, the internal morphology and size of the hydrogel in media with SCF, SGF, and SIF were examined (Figure S8). Our result showed that the cross-sectional morphology appears highly compact at pH 1.2, whereas it becomes more porous and is suitable for drug release in higher pH buffer solutions. The hydrogel particle size increases in the high pH buffer solutions, and surface cracks and collapses begin to appear especially at pH 7.4. This demonstrates that HA-PLGA_bilirubin_ with hydrogel exhibits good pH sensitivity and can protect bilirubin in SGF and subsequently release it in SCF, exhibiting excellent pH sensitivity and controlled-release performance. Furthermore, to evaluate whether HA-PLGA_bilirubin_ improves the targeting ability of bilirubin toward inflamed colon via HA, we investigated the cellular uptake of the nanoparticles by Lipopolysaccharide (LPS)-treated macrophages (*in vitro* inflammatory model). After incubating the cells with free 1,1'-dioctadecyl-3,3,3',3'-tetramethylindodicarbocyanine perchlorate (DID), DID-PLGA_bilirubin_, and DID-HA-PLGA_bilirubin_ for 4 h, fluorescence-activated cell sorter (FACS) analysis was used to determine the intracellular fluorescence intensity of DID (Figure 2B). It was found that the internalization of DID-HA-PLGA_bilirubin_ was approximately 1.69-fold higher than DID-PLGA_bilirubin_ (Figure 2C). Raw 264.7 macrophages treated with DID-HA-PLGA_bilirubin_ exhibited noticeably higher intracellular fluorescence intensity than cells treated with DID-PLGA_bilirubin_ (*p*<0.0001, Figure 2D).

The introduction of HA can potentially modulate the interaction of the nanoparticles with macrophages, altering the uptake dynamics and, consequently, enhancing the fluorescent signal intensity 41. Confocal laser scanning microscopy (CLSM) images also validated the enhanced cellular uptake performance (increasing red fluorescence signals) in HA-PLGA_bilirubin_ compared to PLGA_bilirubin_ (Figure 2E). Furthermore, we have investigated the impact of HA-PLGA_Bilirubin_ treatment on the secretion of cytokines in LPS-treated Raw 264.7 macrophages. Our findings demonstrated a significant upregulation of major pro-inflammatory cytokines (Interleukin-1 Beta (IL-1β), Interleukin-6 (IL-6), Tumor Necrosis Factor Alpha (TNF-α), and Interferon-Gamma (IFN-γ)) induced by LPS treatment. However, this effect was substantially attenuated by HA-PLGA_Bilirubin_ treatment (Figure 2F). These results suggest that HA-PLGA_Bilirubin_ is readily taken up by LPS-activated macrophages and has the capacity to downregulate their pro-inflammatory responses by suppressing the release of pro-inflammatory cytokines.

### Biosafety evaluation of HA-PLGA_bilirubin_

A good biosafety is one of the primary prerequisites for nanomaterials used in biomedical applications. *In vitro*, we have conducted cytotoxicity assays using different nanoparticles, including Bilirubin, HA-PLGA-Lip, PLGA_Bilirubin_, and HA-PLGA_Bilirubin_, at concentrations ranging from 0.1 μg/ml to 100 μg/ml on human intestinal epithelial cells (Caco2) and mouse macrophage cells (Raw 264.7) for a 24 h incubation period. The results of the MTT assay demonstrated that none of the tested nanoparticles exhibited significant cytotoxicity. *In vivo*, we investigated the systemic toxicity of the HA-PLGA_bilirubin_ by blood biochemical index detection and histological assessment of major organs. After various treatment for 3 weeks, function indexes of liver and kidney, such as Alanine Aminotransferase (ALT), Aspartate Aminotransferase (AST), Alkaline Phosphatase (ALP), UREA, and Total Protein (TP), did not change significantly, suggesting that oral administration of the nanoparticles did not impair liver and renal function (Figure S9A-B). Furthermore, there were no significant changes in the level of hematological indexes, such as Red Blood Cells (RBC), Red Blood Cells (WBC), Platelets (PLT), Mean Corpuscular Hemoglobin (MCH), and Mean Corpuscular Volume (MCV) (Figure S9C). Additionally, Hematoxylin and Eosin (HE) staining was used to stain the sections of major organs (heart, liver, spleen, lungs, kidneys) and the histological results revealed no evidence of toxic effects, indicating the high biosafety of HA-PLGA_bilirubin_ (Figure S10).

### *In vivo* biological distribution of HA-PLGA_bilirubin_

**Figure 3:**
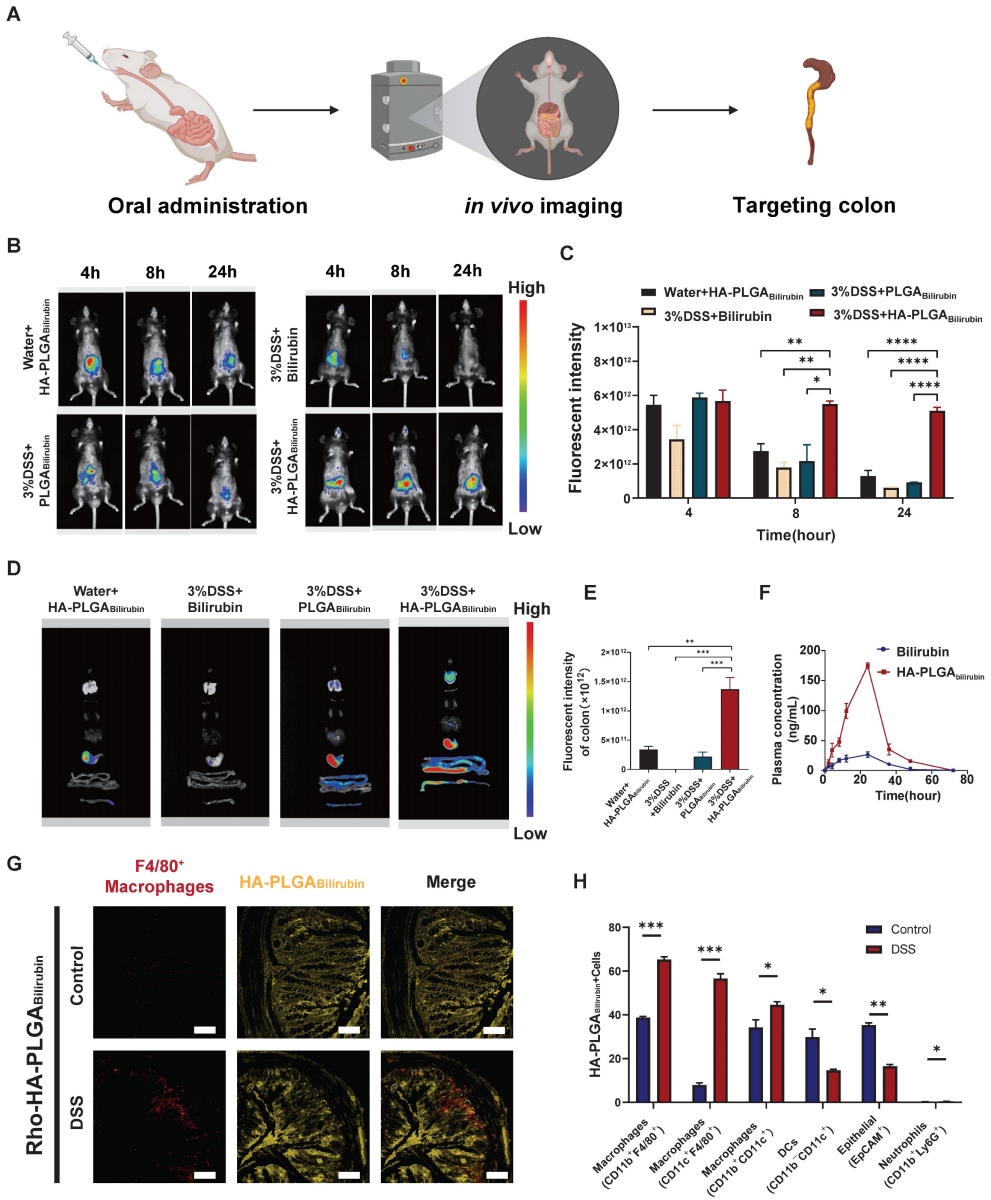
**Orally administered HA-PLGA_bilirubin_ specifically targets the inflamed colon of DSS-induced mice**. (A) Schematic representation of *in vivo* imaging for the DSS-induced mice. (B) Representative images of *in vivo* fluorescence imaging of treating animals with Bilirubin, PLGA_Bilirubin_, or HA-PLGA_Bilirubin_, after 4h, 8h, 24h. (C) Quantification for *in vivo* fluorescence signal at different time-points after various treatment. (D) Representative images of organ fluorescence imaging after 24h. (E) Quantitative fluorescence intensities of the colon. (F) Plasma concentration time profile of free bilirubin and HA-PLGA_Bilirubin_. (G) Representative fluorescence microscopy images of Rho-HA-PLGA_Bilirubin_ uptake in the colon. Scale bar, 50 μm. (H) Targeted cell types in colonic tissues by Cy5.5-HA-PLGA_Bilirubin_. *represents *p*<0.05, **represents *p*<0.01, ***represents *p*<0.001, ****represents *p*<0.0001.

To assess the targeting ability of HA-PLGA_bilirubin_, the accumulation ability of HA-PLGA_bilirubin_ on inflamed intestinal regions was then directly tested, and their biodistribution data was measured using the *In Vivo* Imaging System (IVIS) fluorescence intensities (Figure 3A). Cy5.5 was used to label the free bilirubin and nanoparticles, and normal mice were treated with HA-PLGA_bilirubin_ and Dextran Sulfate Sodium Salt (DSS)-induced mice were treated with bilirubin, PLGA_bilirubin_, and HA-PLGA_bilirubin_. Due to the protective effect of the hydrogel, there were still strong fluorescence signals at 4h in the normal mice and DSS-induced mice treated with PLGA_bilirubin_, and HA-PLGA_bilirubin_, whereas the free DSS-induced mice treated with bilirubin showed decreasing fluorescence. With the extension of time, the fluorescence of normal mice and DSS-induced mice treated with PLGA_bilirubin_ was significantly decreasing, whereas the DSS-induced mice treated with HA-PLGA_bilirubin_ still had a strong fluorescence signal at 12h and 24h. After 24h, DSS-induced mice treated with the same dose of HA-PLGA_bilirubin_ had a significantly higher fluorescence intensity than that of normal mice (~5.46-fold). Furthermore, orally-administered HA-PLGA_bilirubin_ showed at least 8.64-fold and 5.57-fold level of accumulation in DSS-induced mice compared to bilirubin and PLGA_bilirubin_, respectively (Figure 3C). The result of the *ex vivo* fluorescence images of major organs is consistent with the result of *in vivo* images (Figure 3D). Orally-administered HA-PLGA_bilirubin_ exhibited the strongest accumulation (~6.24-fold) in inflame colon tissue compared to PLGA_bilirubin_ (Figure 3E), indicating that PLGA_bilirubin_ bearing HA ligands could significantly enhance the accumulation of bilirubin in the colitis tissues. *In vivo* pharmacokinetic studies further demonstrated that HA-PLGA_bilirubin_ formulation could increase the C_max_ of bilirubin by about 6.69-fold and overall increase in the oral bioavailability by approximately 5.24-fold when compared to the free bilirubin (Figure 3F). Furthermore, we performed fluorescence microscopy on colon tissue sections from DSS-induced colitis mice that had been orally administered Rhodamine (Rho)-HA-PLGA_Bilirubin_. The results demonstrated substantial uptake of the nanoparticles by the inflame colon tissues, and Rho-HA-PLGA_Bilirubin_ exhibited colocalization with macrophages expressing F4/80 (Figure 3G).

Additionally, flow cytometry analysis (Figure 3H; Figure S12) revealed that three major types of macrophages (CD11b^+^CD11c^+^, CD11b^+^F4/80^+^, and CD11c^+^F4/80^+^ cells) exhibited uptake of Cy5.5-HA-PLGA_Bilirubin_, along with dendritic cells (CD11b^-^CD11c^+^ cells; ~14.66%) and epithelial cells (EpCAM^+^ cells; ~16.5%). In contrast, neutrophil uptake (CD11b^+^Ly6G^+^ cells) was minimal (~0.46%). Notably, in the inflamed colon of DSS-induced colitis mice, the uptake of EpCAM^+^ cells for HA-PLGA_Bilirubin_ was significantly reduced compared to the normal colon, suggesting compromised epithelial barrier integrity under colitis conditions. These findings collectively indicate that under conditions of colitis, orally administered HA-PLGA_Bilirubin_ is predominantly taken up by activated macrophages in the inflamed colon exhibiting impaired epithelial barrier function.

### Effective recovery of the morphology and function of the inflamed colon by HA-PLGA_bilirubin_

**Figure 4:**
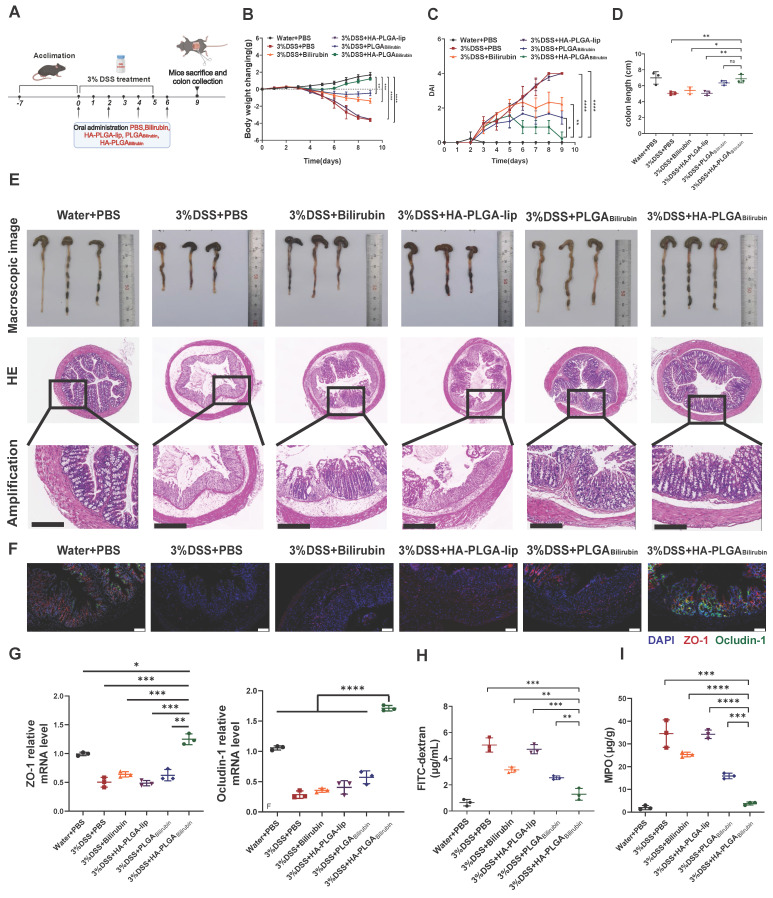
*** In vivo* therapeutic efficacy and recovery of impaired intestinal barrier of HA-PLGA_bilirubin_ in the mice with DSS-induced ulcerative colitis.** (A) Schematic illustration presenting the treatment regime of the DSS-induced ulcerative colitis. Mice were fed with drinking water or drinking water containing 3% DSS for 5 days. On days 0, 2, 4, 6, mice were treated with PBS or 30 mg kg^-1^ of Bilirubin, HA-PLGA-lip (with 100K HA at equivalent mass), PLGA_Bilirubin_ (with 30 mg kg^-1^ bilirubin at equivalent mass), HA-PLGA_Bilirubin_ (with 100K HA and 30 mg kg^-1^ bilirubin at equivalent mass) and mice were subsequently sacrificed at day 9. (B) Body weight changes of the mice for 9 days. (C) Disease activity index (DAI) of the mice for 9 days. (D) Statistic colon length at day 9. (E) The representative microscopic and the representative HE staining image of colon tissue after various treatment. Scale bar, 250 μm. (F) Fluorescence microscopy depicting the expression levels of ZO-1 and Ocludin-1 in the colon tissues. Scale bar, 100 μm. (G) mRNA expression levels of ZO-1 and Ocludin-1 in colon tissues. (H) FITC-dextran assay for measuring intestinal permeability. (I) MPO level in the colon tissues after various treatment. *represents *p*<0.05, **represents *p*<0.01, ***represents *p*<0.001, ****represents *p*<0.0001. ns: not statistically.

To assess the therapeutic effect of HA-PLGA_bilirubin_ against colitis, DSS-induced mice were treated with PBS, free bilirubin (30 mg/kg) and corresponding dose of various nanoparticles formulations the oral route on days 0, 2, 4 and 6 42 (Figure 4A). Compared with PBS and other treatment groups, the DSS-induced mice given HA-PLGA_bilirubin_ significantly prevented animals from bodyweight loss (Figure 4B), lower Disease Activity Index (DAI) values (Figure 4C), and shorter colon lengths (Figure 4D). Next, HE staining was used to stain colon tissue samples treated with different groups to evaluate the histological damages (Figure 4E). Compared with control health mice, DSS-induced mice showed significantly larger inflammation, characterized by depletion of goblet cells, mucosal thickening, colonic epithelium damage, inflammatory cell infiltration. Obviously, HA-PLGA_bilirubin_ treatment showed significantly reduction of these inflammatory appearances than all other treatment groups and was similar to the healthy control group. Notably, histological damage of HA-PLGA-lip (empty biomaterial without bilirubin) group was similar to that in DSS-induced mice, indicating HA-PLGA_bilirubin_ may exhibit their effects via increasing the accumulation of bilirubin and not HA or PLGA. DSS-induced mice treated with PLGA_bilirubin_ did not reach full histological recovery, suggesting HA contributed to the retention and therapeutic efficacy of bilirubin in the inflame colon. Given that DSS-induced colitis is known to cause disruptions in intestinal tight junctions and barrier integrity, we evaluated changes in the expression of tight junction-related proteins, such as ZO-1 and Ocludin-1. Immunohistochemistry (IHC) and mRNA expression analyses were performed, and the results (Figure 4F-G) revealed significant reductions in the levels of these proteins in the colonic tissue of mice treated with PBS and HA-PLGA-lip, as expected. While bilirubin and PLGA_bilirubin_ treatments demonstrated slight restoration of these proteins, the treatment with HA- PLGA_bilirubin_ significantly restored the levels of ZO-1 and Ocludin-1 in the colon, bringing their protein and mRNA levels back to levels similar to those in the normal control group. In terms of intestinal permeability, we assessed this by monitoring serum fluorescence intensity following the oral administration of fluorescein isothiocyanate-labeled dextran (FITC-dextran). As anticipated, the HA-PLGA_bilirubin_ treated group exhibited the lowest serum fluorescence intensity among all treatment groups (Figure 4H), indicating the lowest intestinal permeability. On the other hand, MPO level were the crucial indicators to evaluate the inflammation function repair of ulcerative colitis. As shown in Figure 4I, HA-PLGA_bilirubin_ treatment decreased the level of MPO activity than other treatment (*p*<0.001). Together, all these findings demonstrated that HA-PLGA_bilirubin_ exhibited effective recovery of the bowel's morphology and intestinal barrier function.

### Promotion of colonic epithelial/stem cells regeneration after treatment with HA-PLGA_bilirubin_

**Figure 5:**
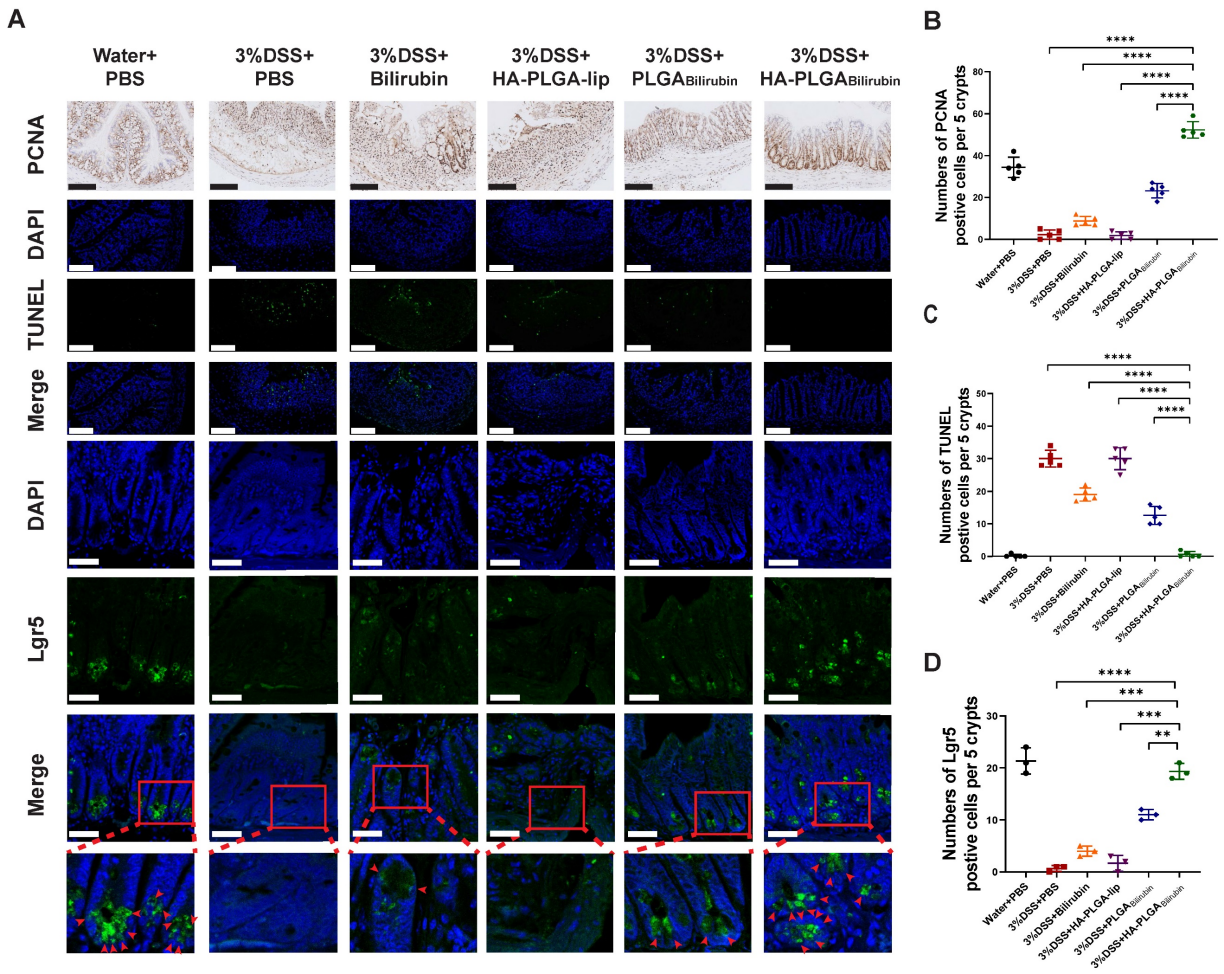
** Effects of HA-PLGA_bilirubin_ on inflamed colon cell proliferation, apoptosis and stem cell regeneration.** (A) Representative Immunohistochemical images for PCNA staining (Scale bar, 140 μm), and the representative immunofluorescence images for TUNEL staining (Scale bar, 140 μm) and Lgr5 staining (Scale bar, 50 μm). (B) Quantitative analysis of the immunohistochemical staining of PCNA. (C) Quantitative analysis of the immunofluorescence staining of TUNEL. (D) Quantification of Lgr5 immunofluorescent staining. *represents *p*<0.05, **represents *p*<0.01, ***represents *p*<0.001, ****represents *p*<0.0001.

Modulation of epithelial proliferation/apoptosis, and stem cell regeneration were the crucial processes maintaining the homeostasis of intestine epithelial system 43, 44. In this study, Proliferating Cell Nuclear Antigen (PCNA) and Terminal deoxynucleotidyl transferase dUTP Nick End Labeling (TUNEL) staining were used to evaluate proliferation and apoptosis of intestine epithelial cells. Compared with other treatment, HA-PLGA_bilirubin_ treatment did significantly promote colonic epithelial proliferation and reduce apoptosis (Figure 5A-C). Fast-cycling Lgr5^+^ intestinal epithelial stem cells (IESC) appear to support colonic epithelium regeneration 32, which may result in the renew and recovery of epithelium after intestinal inflammation. Herein, we used immunofluorescence staining for Lgr5 to quantify Lgr5 expression and localize lgr5^+^IESC. As expected, DSS-induced mice and HA-PLGA-lip group both had a visible loss of IESC density in the colonic crypts (Figure 5A). Obviously, a significant higher density of Lgr5^+^positive IESC was observed in the intestinal crypts of HA-PLGA_bilirubin_ and PLGA_bilirubin_ treatments compared to other groups (Figure 5D). Moreover, DSS-induced mice given HA-PLGA_bilirubin_ showed a more drastic proliferation of IESC in the colonic crypts than that of DSS-induced mice treated with PLGA_bilirubin_, indicating the optimal stem cell regeneration performance of HA-PLGA_bilirubin_.

### Alleviation of the angiogenesis and inflammation after treatment with HA-PLGA_bilirubin_

**Figure 6:**
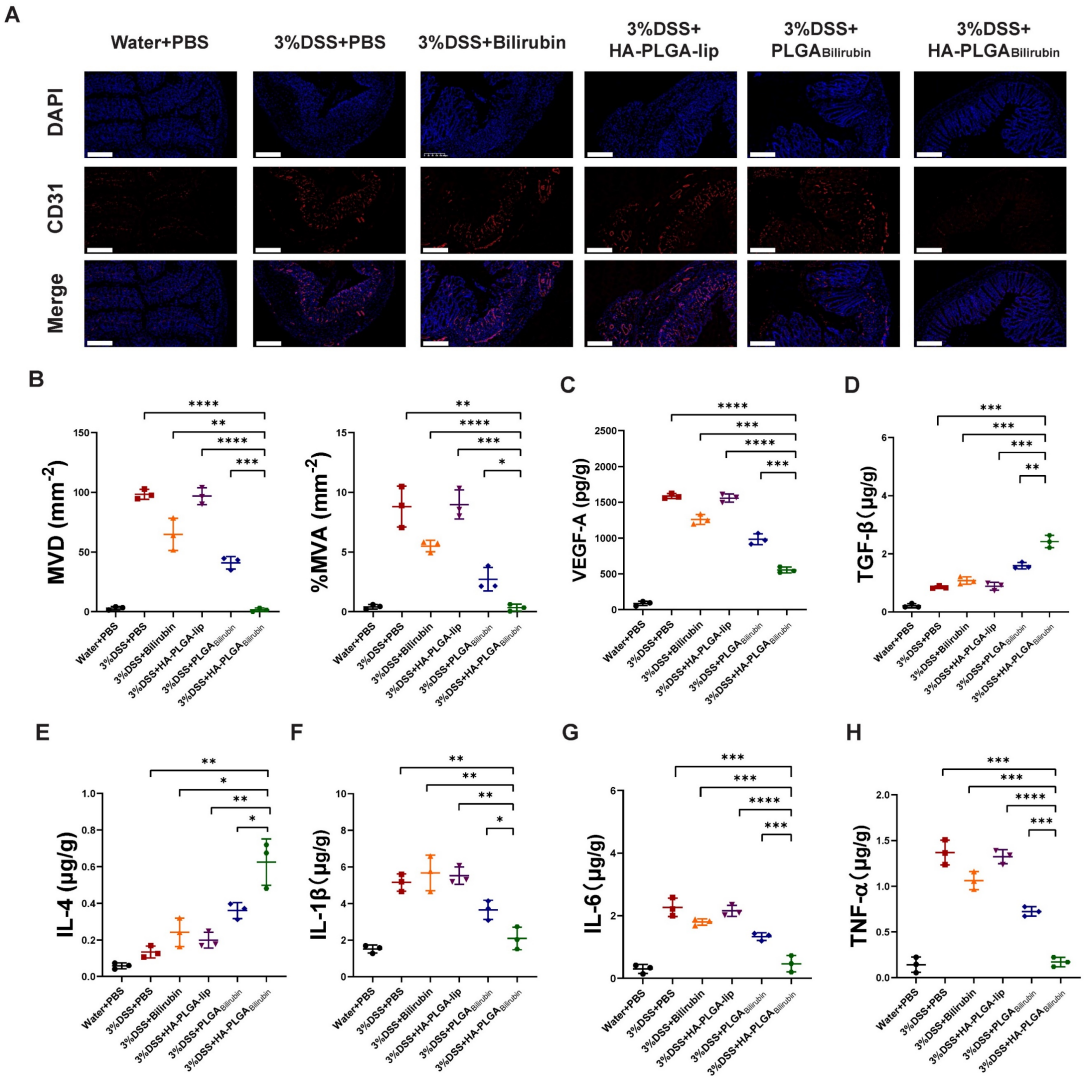
** HA-PLGA_bilirubin_ against ulcerative colitis via alleviating angiogenesis and inflammation of inflamed colon**. (A) Representative immunofluorescence images for CD31 staining after various treatment. Scale bar, 240 μm. (B) The mean vessel density (MVD) and mean vessel area/mm^2^ (MVA) of mucosa were quantified. (C) VEGF-A level in the colon tissues after various treatment. (D, E, F, G, H) Inflammatory factor level of TGF-β (D), IL-4 (E), IL-1β (F), IL6 (G), and TNF-α (H) in the colon tissues after various treatment. *represents *p*<0.05, **represents *p*<0.01, ***represents *p*<0.001, ****represents *p*<0.0001.

Massive angiogenesis cascade strongly correlated with the progression of experimental colitis 45. Herein, the reduction of angiogenesis was assessed by CD31 (an endothelial marker) staining and vascular endothelial growth factor A (VEGF-A) expression level. As shown in Figure 6A, the amount of CD31 positive cells in inflamed mucosa of DSS-induced mice was significantly higher than in control healthy mice, which is consistent with previous study 45. Conversely, HA-PLGA_bilirubin_ treatment significantly decreased the mean vessel density and area, as well as VEGF-A level (Figure 6B-C) in the colon mucosa than that of PLGA_bilirubin_ treatment. On the other hand, Enzyme-Linked Immunosorbent Assay (ELISA) method was used to determine inflammatory factors measurement of tissue homogenate. HA-PLGA_bilirubin_ treatment significantly increased the level of anti-inflammatory cytokines of Transforming Growth Factor Beta (TGF-β) and Interleukin-4 (IL-4) (Figure 6D-E), and reduce the level of pro-inflammatory cytokines of IL-1β (Figure 6F), IL-6 (Figure 6G), TNF-α (Figure 6H) compared to DSS-induced mice and HA-PLGA-lip. Among three bilirubin formulations, HA-PLGA_bilirubin_ treatment displayed the most obvious amelioration on inflammation and angiogenesis, suggesting that the assembly of HA and PLGA could enhance the potential effect of bilirubin on DSS-induced colitis.

### HA-PLGA_bilirubin_ treatment reprograms transcriptional landscape *in vivo*

**Figure 7:**
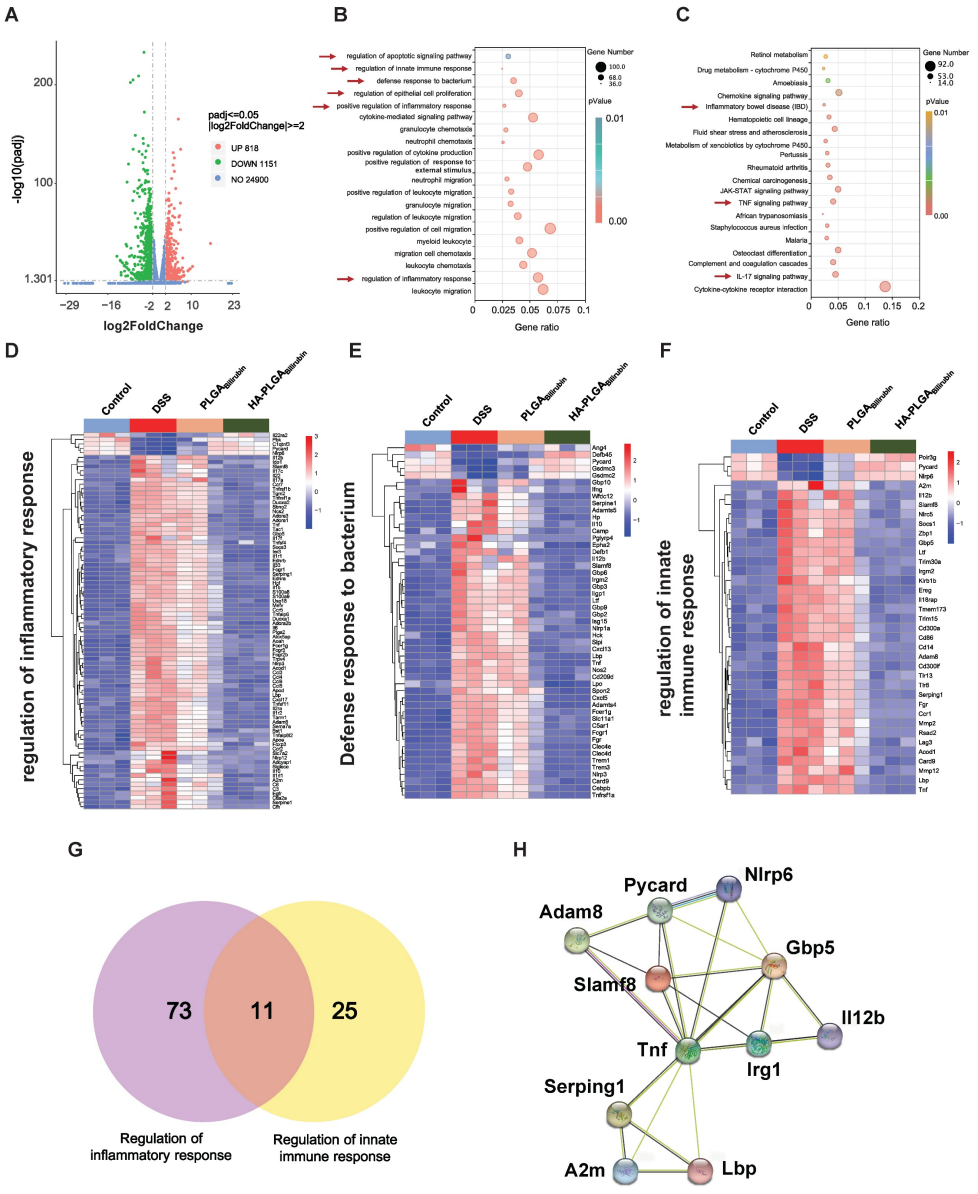
** Transcriptomic analysis of the colon tissues.** (A) A Volcano plot presenting the significantly differentially expressed genes (*p*<0.05 and |Log_2_ Fold change| ≥2) between DSS+HA-PLGA_bilirubin_ group compared to the DSS group, including 818 upregulated genes and 1,151 downregulated genes. (B, C) GO enrichment analysis (B) and KEGG pathway analysis (C) of the significantly differentially expressed genes. (D, E, F) The heatmap of differentially expressed genes associated with regulation of inflammatory response (D), defense response to bacterium (E) and regulation of innate immune response (F). (G) Venn diagram of the differentially expressed genes between comparisons. 11 genes regulate both regulation of inflammatory response and regulation of innate immune response. (H) Functional association networks of the co-expressed genes that allow the simultaneous regulation of both regulation of inflammatory response and regulation of innate immune response.

To further demonstrate the recovery mechanism of HA-PLGA_bilirubin_ *in vivo*, colon tissues were harvested from control healthy mice and DSS-induced mice treated with PBS, PLGA_bilirubin_, HA-PLGA_bilirubin_ for "transcriptomic analyses. A total of 1,969 genes was differentially expressed between DSS-induced mices given PBS and HA-PLGA_bilirubin_ (*p*_adjust_<0.05, |log2FoldChange|≥2), of which 818 genes were upregulated and 1,151 genes were downregulated (Figure 7A). Significant differences existed between the groups in terms of the expression level of these 1,969 genes (Figure S13A). Gene Ontology (GO) enrichment analysis showed that these Differentially Expressed Genes (DEGs) were mainly involved in the regulation of inflammatory response, regulation of epithelial cell proliferation, defense response to bacterium, regulation of innate immune response, and regulation of apoptotic signaling pathway (Figure 7B). In the Kyoto Encyclopedia of Genes and Genomes (KEGG) pathway analysis, enriched pathways and diseases included inflammatory bowel disease (IBD), TNF-signaling pathway, and IL-17 signaling pathway (Figure 7C; Figure S13B--C). More specifically, upregulated genes were mainly associated with the metabolism modulation, including fatty acid metabolic process, retinol metabolism, and acid metabolism (Figure S14A-B).

The downregulated genes were mainly involved in the regulation of inflammatory response, positive regulation of defense response, Toll-like receptor signaling pathway, and NOD-like receptor signaling pathway, which played a key role in the inflammatory and immune responses (Figure S14C-D). Moreover, we further analyzed the function of innate immunity and host defense-related genes, and gene set with specific functions of each group indicated that regulation of inflammatory response-related genes (Figure 7D), defense response to bacterium-related genes (Figure 7E), and regulation of innate immune response-related genes (Figure 7F) were obviously downregulated in HA-PLGA_bilirubin_ treatment. As inflammation and immune regulation are highly associated with UC recovery 46, we obtained 11 genes that participated in the regulation of inflammatory response and regulation of innate immune response together after HA-PLGA_bilirubin_ treatment (Figure 7G). A PPI analysis revealed significantly enriched interactions between those genes (Figure 7H). Furthermore, we have included Western blot experiments to investigate the expression levels of key proteins involved in the TNF and Interleukin-17 (IL-17) signaling pathways, such as TNF-ɑ and IL-17A, providing additional evidence for the therapeutic role of HA-PLGA_Bilirubin_ hydrogels. Our results show that TNF-ɑ and IL-17A protein levels are significantly increased during the inflammatory process, and HA-PLGA_Bilirubin_ hydrogels can significantly reduce the expression of these two proteins (Figure S15), indicating that HA-PLGA_Bilirubin_ hydrogel may repair DSS-induced colitis by negatively regulating the TNF and IL-17 signaling pathways. Together, HA-PLGA_bilirubin_ treatment significantly elicit a regulation of inflammatory response and innate immune response to repair DSS-induced colitis.

## Discussion

To our knowledge, our study is the first attempt to combine the pH-responsive hydrogels with HA-based bilirubin for the dual-targeting treatment of UC. Compared with previous studies which suffer from HA's susceptibility to diffusion or degradation in digestive juice 18, 19, our proposed nanoparticles overcome these dilemmas, and dramatically improve the release and accumulation of bilirubin at the inflammatory location of UC through pH-responsive reaction and HA-targeted inflammatory macrophages mechanism. Moreover, our *in vivo* experiments support the potent therapeutic efficacy of HA-PLGA_bilirubin_ and show some novel recovery mechanisms associated with intestinal epithelial/stem cell regeneration and immune response modulation capabilities.

The study has certain strengths. First, we have enhanced the oral applicability of traditional HA-bilirubin nanoparticles by introducing pH-responsive hydrogels. In the previous study, Lee et al. and Huang et al. have designed HA-bilirubin nanoparticles through amphiphilic conjugation effects between HA and bilirubin 18 or self-assembly of ε-polylysine-bilirubin and HA 19. However, HA-based nanoparticles may suffer from their uncontrollable systemic diffusion and adverse systemic effects during oral administration delivery to treat colon disease 47. To eliminate the impact of HA, we embedded them into a pH-responsive hydrogel, and this binding may protect the HA-based bilirubin from premature degradation and bursting release by gastric proteolytic enzymes. As expected, our study showed that hydrogels may not only protect HA-based bilirubin from premature degradation by gastric enzymes, but also allows HA-based bilirubin to be targeted to specific sites in the gastrointestinal tract without premature release. Therefore, combination of hydrogels and HA-bilirubin nanoparticles may be a highly effective approach for accelerating the healing process of ulcers in UC mice.

Second, we further investigated and observed the therapeutic effects and mechanisms of HA-PLGA_bilirubin_ hydrogels in UC. Previous studies have reported that bilirubin exerts antioxidative, anti-inflammatory, and immunoregulatory effects against various inflammatory diseases 16, 48-50. However, these studies on the mechanisms underlying the therapeutic effects of bilirubin in UC have been relatively superficial and require to be further clarified. In our experiments, the prepared bilirubin nanoparticles were found to be able to repair damaged colon in UC with the superior therapeutic effects compared to bilirubin therapy alone. Further experiments revealed that such beneficial effects were associated with the modulation of intestinal epithelial/stem cells regeneration and decreasing of angiogenesis and inflammation. In particular, this is also the first demonstration that HA-PLGA_bilirubin_ treatment can ameliorate DSS-induced colitis by modulating colonic epithelial/stem cells regeneration, which extends our knowledge on the therapeutic mechanisms of bilirubin 51-53. By shedding light on this innovative area of research, our findings may pave the way for the development of bilirubin for the treatment of chronic inflammatory conditions.

At last, we explored the potential immune mechanism of HA-PLGA_bilirubin_ hydrogels in recovering mucosal damage in UC. Previous studies indicated that innate immune responses played a vital role in the development and protection of UC 54-58. Similarly, we uncovered the significant alteration of innate immune responses before and after HA-PLGA_bilirubin_ treatment using transcriptome profiling 59. This alteration was especially related to TNF and IL-17 signaling pathway, which have been reported to be critically involved in the pathogenesis of various inflammatory disorders including UC 60-62. Herein, our findings demonstrated that HA-PLGA_bilirubin_ hydrogels may play a therapeutic role by modulation of innate immune responses especially negative regulation of TNF and IL-17 signaling pathway. As UC is a polygenic disease related to immunodeficiency 63, 64, we further screened 11 genes including TNF that interact with it, which may provide potential targets of intervention for UC.

## Conclusions

To sum up, our work developed a novel bilirubin delivery system that formed by hydrogel encapsulating hyaluronic acid (HA)-functionalized PLGA/liposome nanoparticles (HA-PLGA_bilirubin_). The prepared nanoparticles exhibit potent pH-responsive delivery and HA-targeting functions, which enhances the repair effects of bilirubin via modulation of intestinal epithelial/stem cells regeneration and immune response. It is envisaged that this study will provide a safe and promising oral drug delivery system for the treatment of ulcerative colitis.

## Supplementary Material

Supplementary figures and table.
