# Supplementary material for: Targeted modulation of intestinal epithelial regeneration and immune response in ulcerative colitis using dual-targeting bilirubin nanoparticles
Source: Theranostics. 2024 Jan 1;14(2):528–46. doi: 10.7150/thno.87739 (PMC10758062; doi:10.7150/thno.87739)
Supplement: Supplementary file 1 — Supplementary figures and table. [file thnov14p0528s1.pdf]

SUPPLEMENTAL INFORMATION

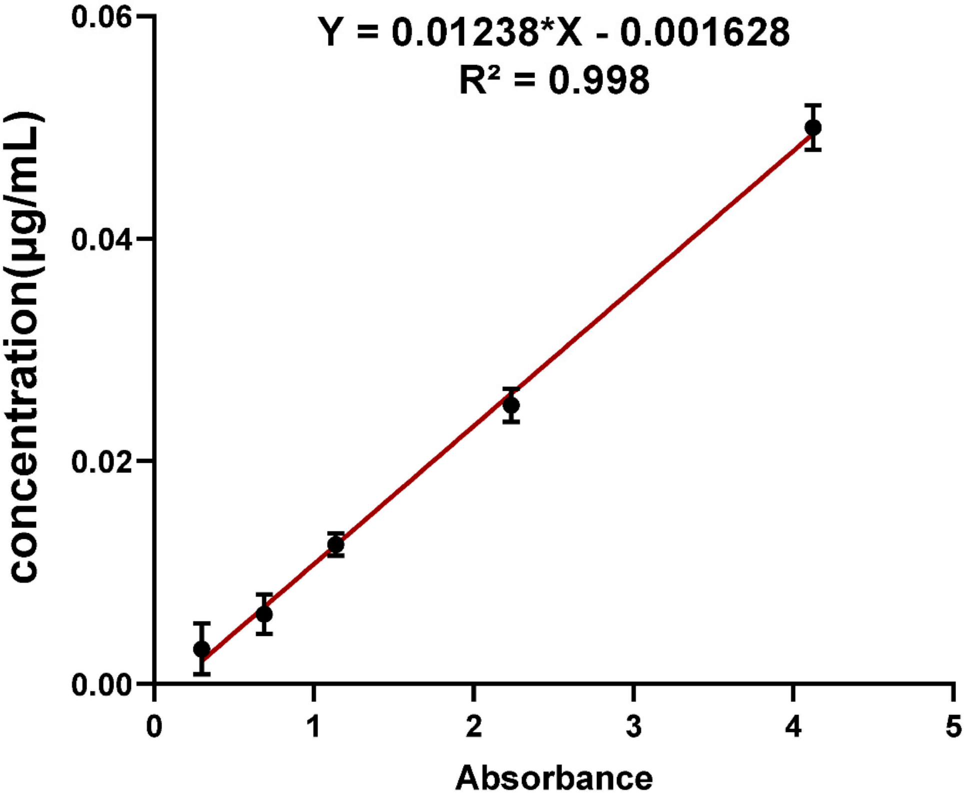

Supplementary Figure 1. The standard curve between the bilirubin concentration and absorbance.

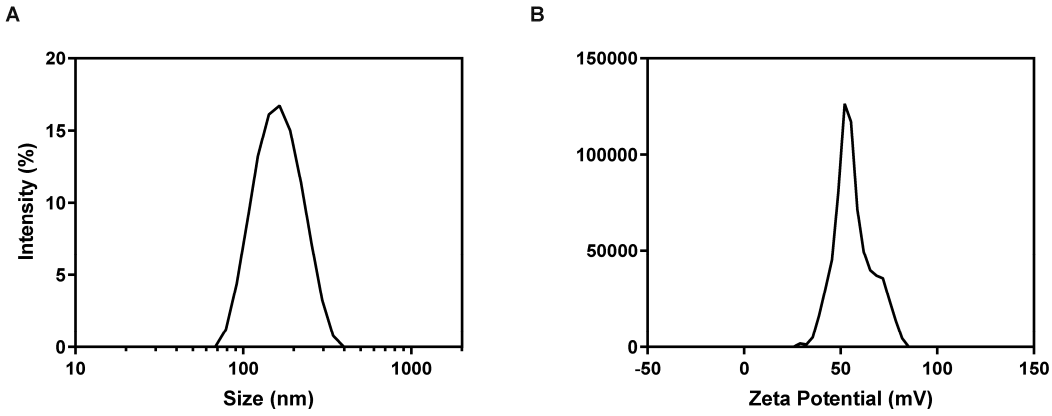

Supplementary Figure 2. Preparation of PLGA$_{bilirubin}$ nanoparticles. (A) Size distribution of PLGA$_{bilirubin}$. The size of PLGA$_{bilirubin}$ was stable at 160 nm. (B) Zeta potential of PLGA$_{bilirubin}$. The zeta potential of PLGA$_{bilirubin}$ was stable at 55mV.

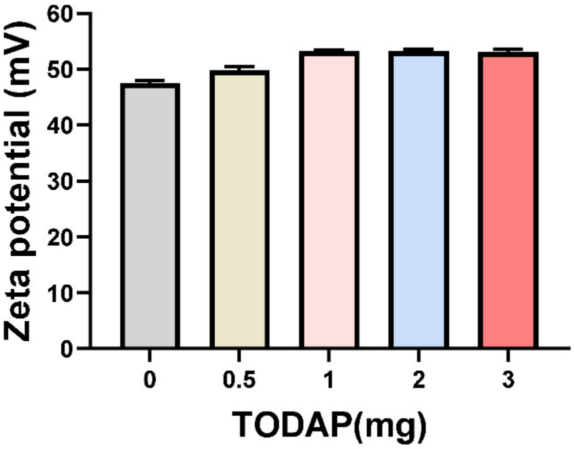

Supplementary Figure 3. **The change of zeta potential with the increasing DOTAP.**

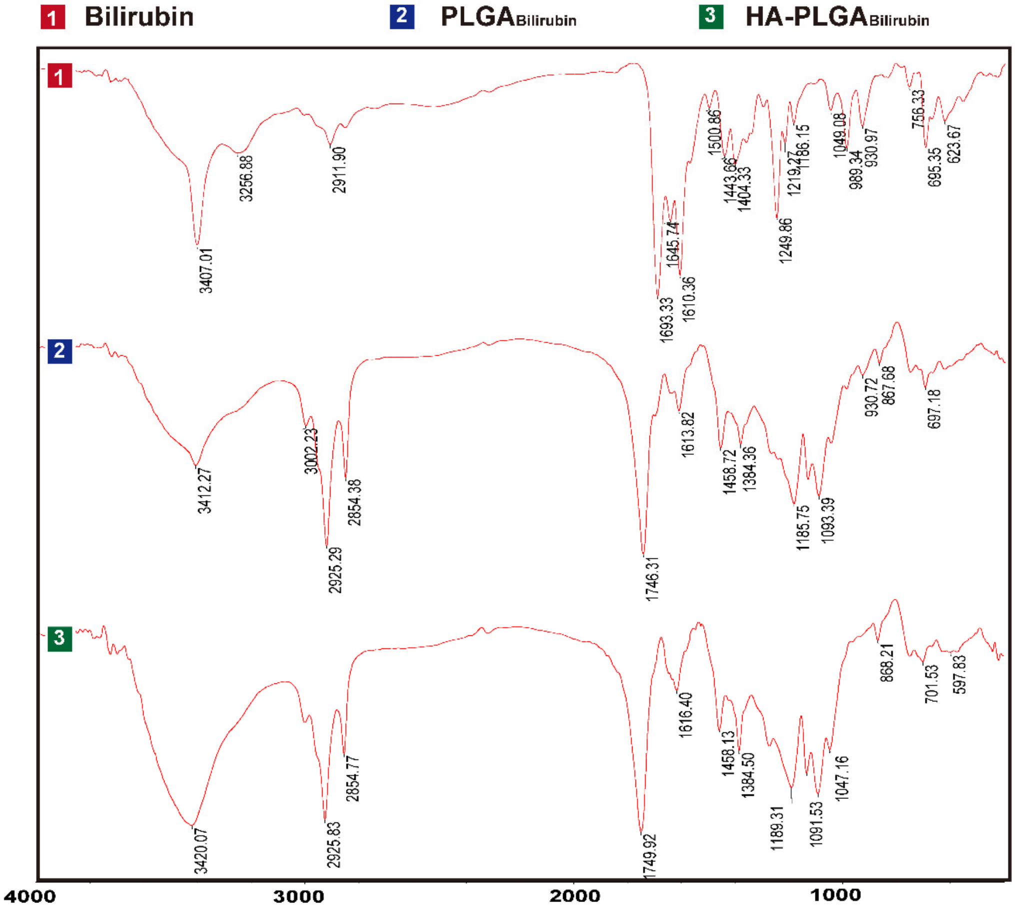

Supplementary Figure 4. The FT-IR spectra of Bilirubin, PLGA$_{Bilirubin}$, and HA-PLGA$_{Bilirubin}$ revealed significant structural information.

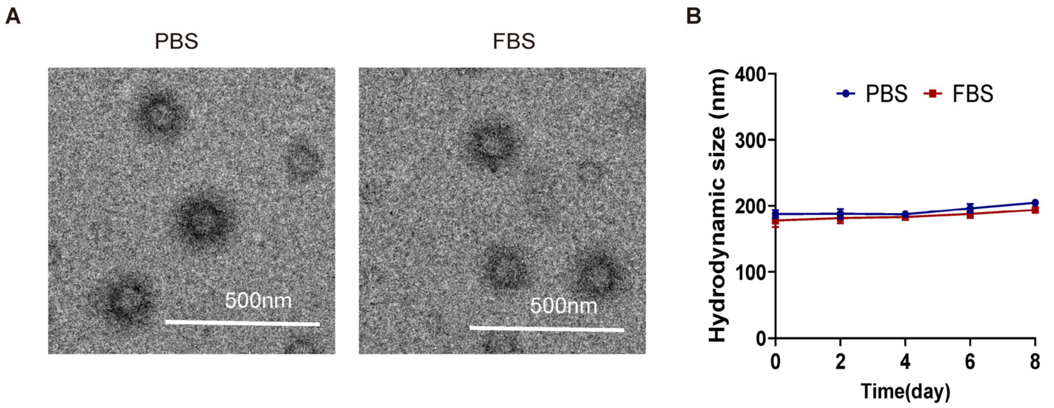

Supplementary Figure 5. Stability of HA-PLGA$_{Bilirubin}$ nanoparticles. (A) TEM images of HA-PLGABilirubin in PBS and FBS. (B) The hydrodynamic size of HA-PLGA$_{Bilirubin}$ in PBS and FBS.

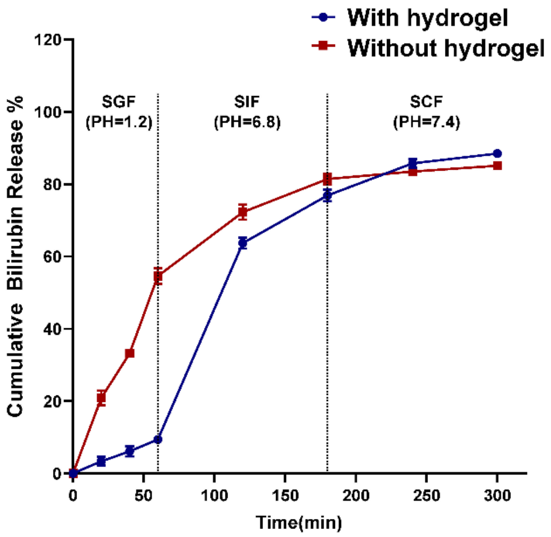

Supplementary Figure 6. Effect of the HA-PLGA$_{Bilirubin}$ with or without hydrogel on the amount of Bilirubin release in different digestion solutions.

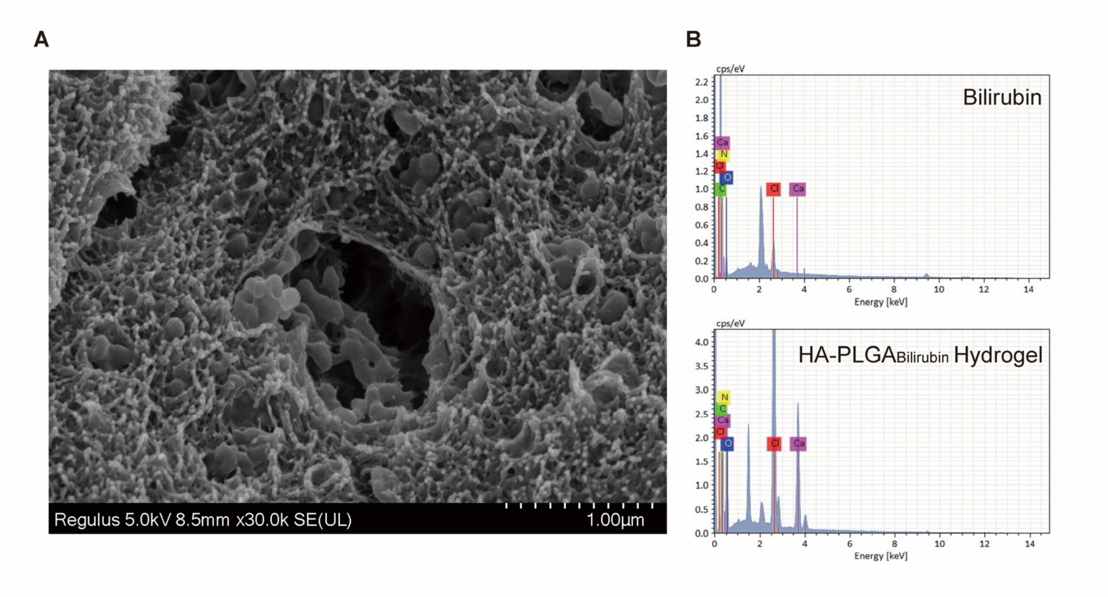

Supplementary Figure 7. SEM-EDS analysis of HA-PLGA$_{Bilirubin}$. (A) SEM images of the HA-PLGA$_{Bilirubin}$. (B) EDS results of bilirubin before and after Hydrogel adding.

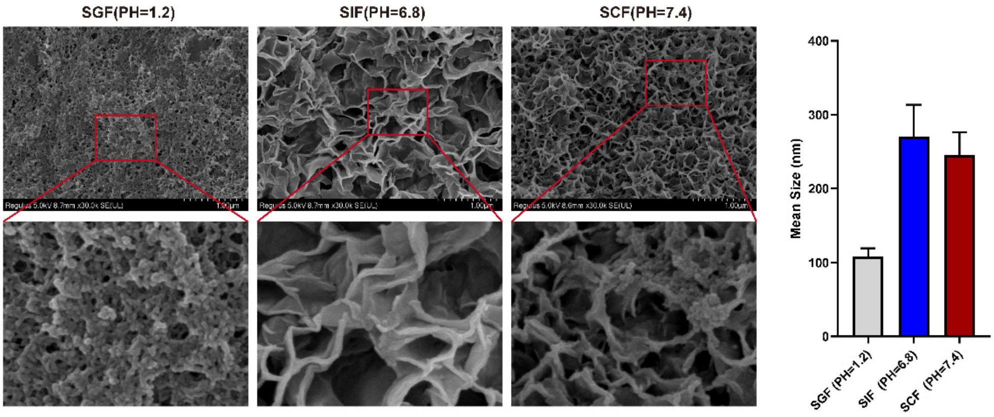

Supplementary Figure 8. The SEM photographs and mean size of HA-PLGABilirubin hydrogel at SGF (PH=1.2), SIF (pH 6.8), and SCF (pH 7.4).

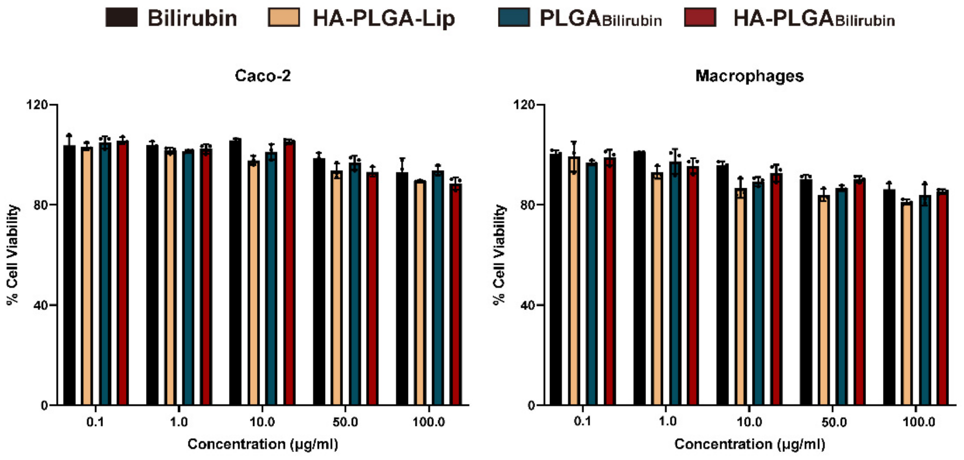

Supplementary Figure 9. Cytotoxicity studies of different nanoparticles on colon epithelial-like cells (Caco-2) and macrophages (Raw 264.7).

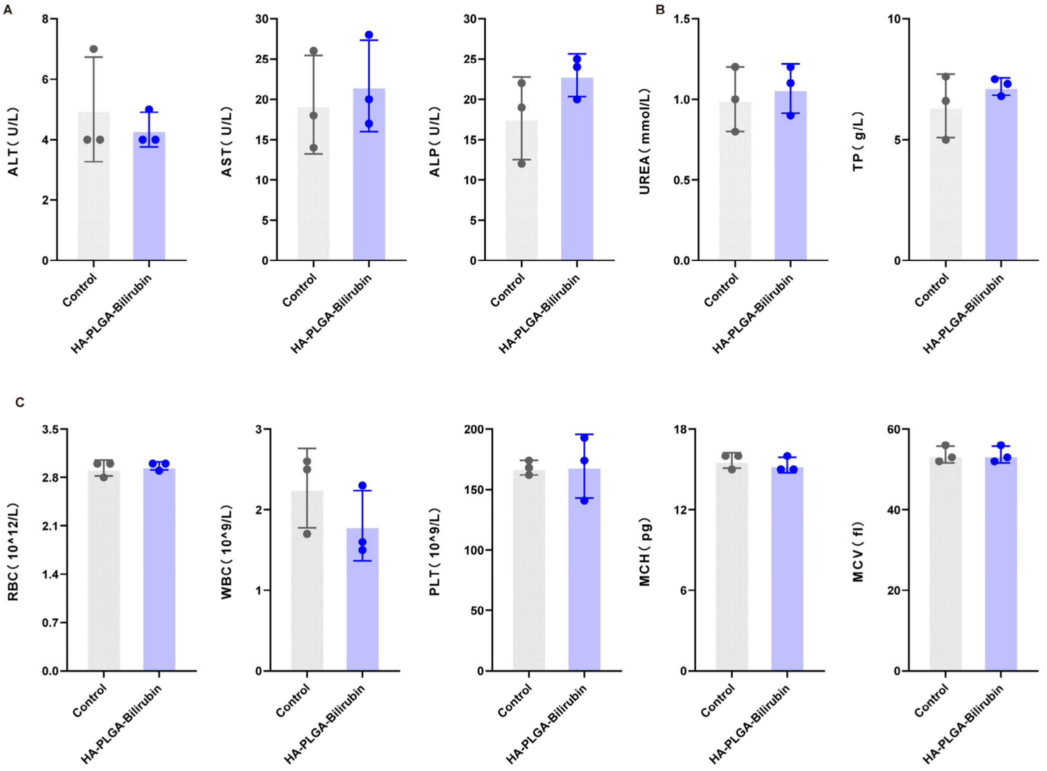

Supplementary Figure 10. Serum biochemistry and hematological indexes of HA-PLGA$_{\mathbf{bilirubin}}$. (A) Function indexes of liver (ALT, AST, ALP). (B) Function indexes of kidney (UREA, TP). (C) Hematological indexes (RBC, WBC, PLT, MCH, MCV)

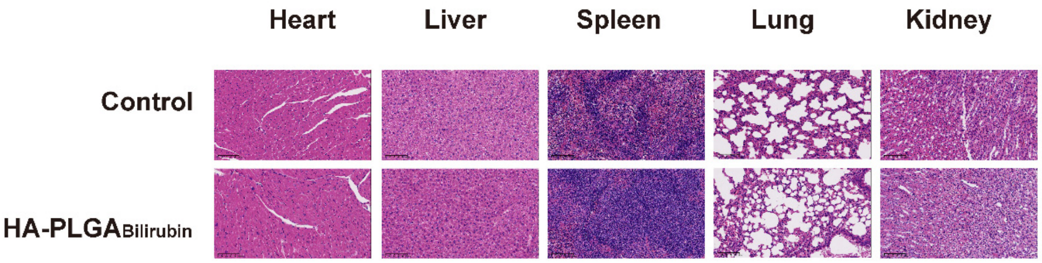

Supplementary Figure 11. Representative HE-stained sections of heart, liver, spleen, lung, and kidneys of mice treated with saline (control) or HA-PLGA$_{\text{bilirubin}}$.

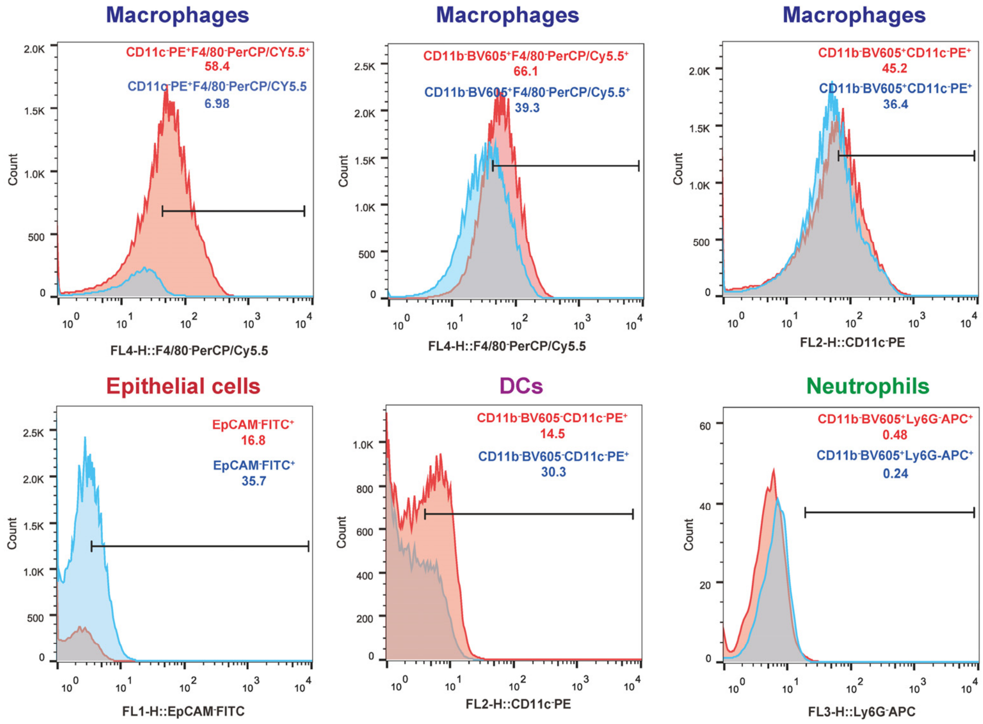

Supplementary Figure 12. Flow cytometry analysis of the percentage of Cy5.5-HA-PLGA$_{\text{Bilirubin}}^{+}$ cells among different cell subtypes in both normal and DSS-induced colitis mice.

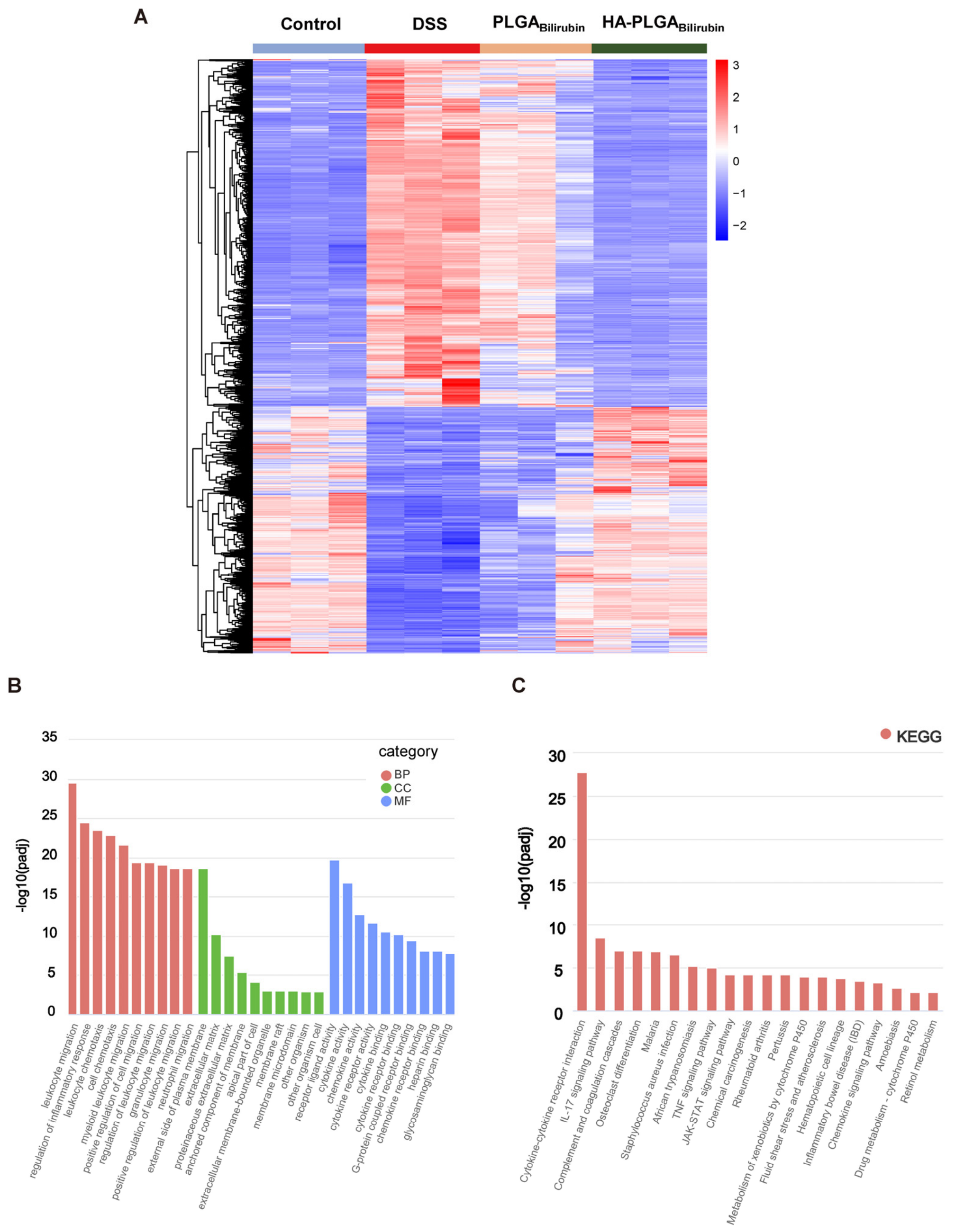

Supplementary Figure 13. (A) Differentially expressed genes of the colon tissues after treatment with PBS, DSS, DSS+PLGA$_{bilirubin}$, DSS+HA-PLGA$_{bilirubin}$. (B, C) GO enrichment (B) and KEGG analysis (C) of the differentially expressed genes after treatment with PBS and DSS+HA-PLGA$_{bilirubin}$.

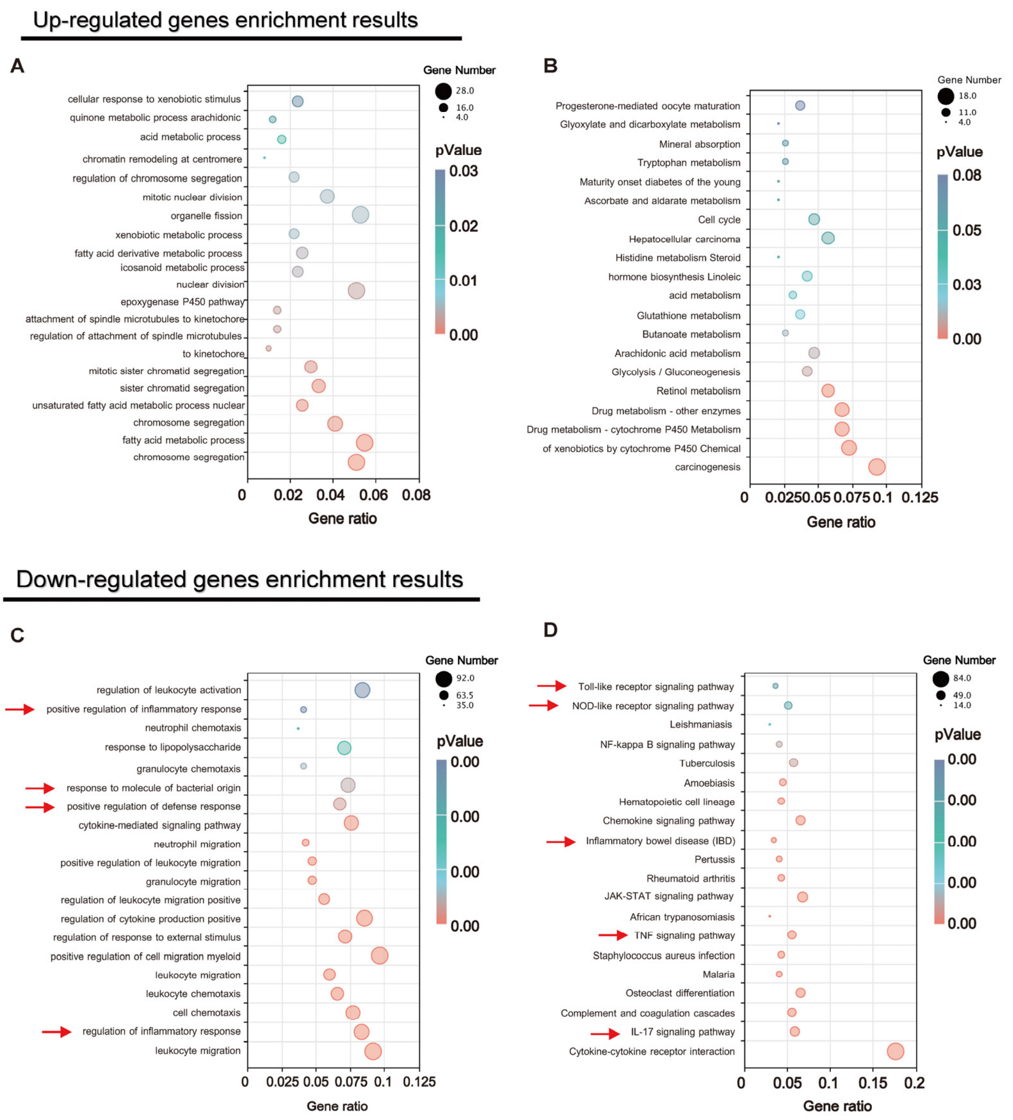

Supplementary Figure 14. GO and KEGG analysis of differential expressed genes. (A, B) GO (A) and KEGG (B) analysis of the up-regulated genes in DSS+HA-PLGA$_{bilirubin}$ group compared to the DSS group. (C, D) GO (C) and KEGG (D) analysis of the down-regulated genes in DSS+HA-PLGA$_{bilirubin}$ group compared to the DSS group.

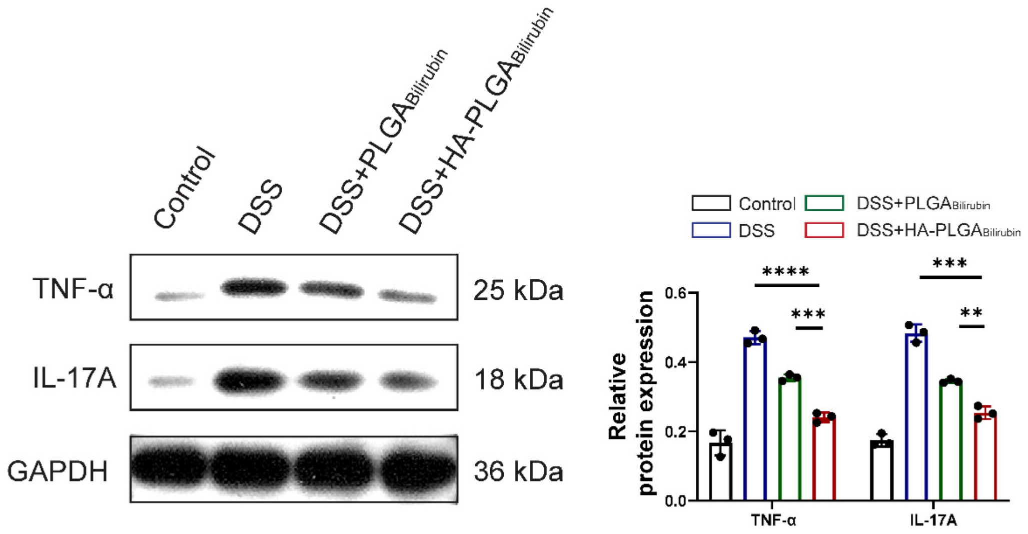

Supplementary Figure 15. Relative protein expression of TNF-α and IL-17A.

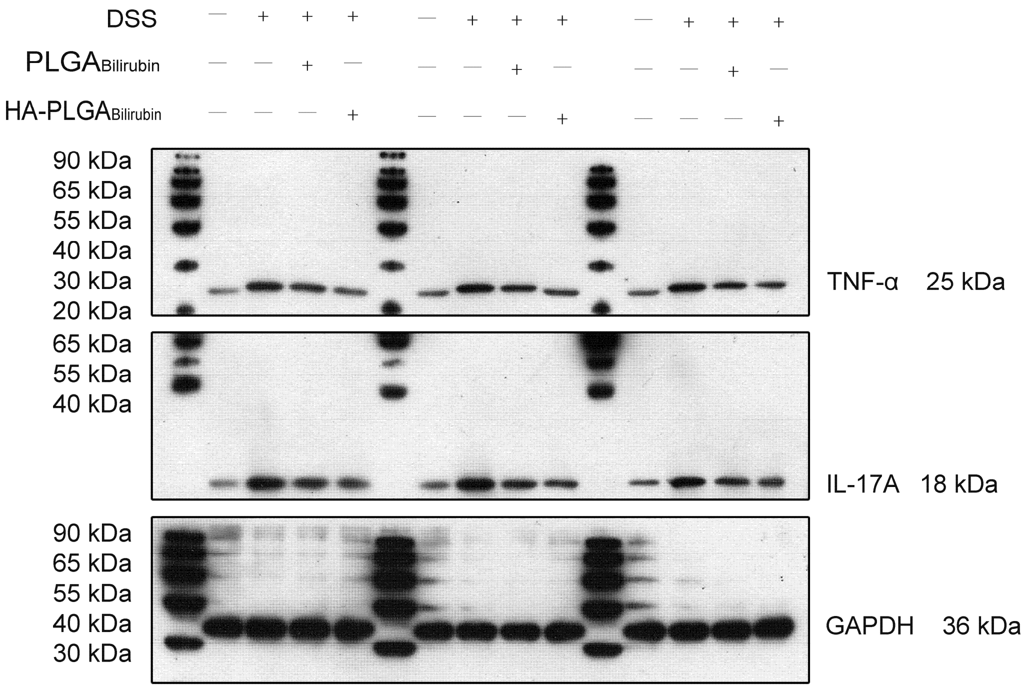

Supplementary Figure 16. Full-length Western blots.

Table S1. Physicochemical characteristics of the Bilirubin-loaded nanoparticles (mean ± S.E.M.; n=3).

NPs	$PLGA_{Bilirubin}$	$HA\text{-}PLGA_{Bilirubin}$
Particle size (nm)	156.83±17.23	193.23±12.85 nm
PDI	0.114±0.006	0.192±0.004
Zeta potential (mV)	49.71±1.63	24.86±2.55
